# Evolution of Sexual Dimorphism in Tube Blennies (Teleostei: Chaenopsidae)

**DOI:** 10.1093/iob/obz003

**Published:** 2019-03-06

**Authors:** Philip A Hastings

**Affiliations:** Marine Biology Research Division, Scripps Institution of Oceanography, University of California San Diego, 9500 Gilman Drive, La Jolla, CA 92093-0244, USA

## Abstract

The study of sexual differences provides insights into selective factors operating on males and females, especially for clades exhibiting varied levels of dimorphism. Sexual differences in morphology and coloration (melanophores) were compiled for 66 of the 89 species of tube blennies (Blenniiformes, Chaenopsidae) from the systematic literature and examination of preserved specimens. Chaenopsids include essentially monomorphic species and those in which males and females differ in as many as 17 morphological and 14 coloration features. While the sexes of most species differ in coloration (at least at the time of breeding), they are morphologically similar in *Acanthemblemaria*, *Hemiemblemaria*, and *Lucayablennius*. While other genera exhibit an intermediate level of dimorphism, species of *Coralliozetus*, *Cirriemblemaria*, and *Emblemaria* are dramatically dimorphic. Character maps on a phylogenetic hypothesis indicate that this extreme level of dimorphism evolved independently in these genera. A complex history of evolution is implied by examination of jaw length with both increases and decreases in one or both sexes leading to either dimorphism or monomorphism. Several features related to shelter defense are monomorphic in species where both sexes inhabit shelters, but dimorphic where only males occupy shelters. Other dimorphic features increase the conspicuousness of male courtship and aggressive displays.

## Synopsis

Translations: Evolution des sexuellen Dimorphismus bei Hechtschleimfischen (Teleostei: Chaenopsidae) (Evolution of sexual dimorphism in tube blennies [Teleostei: Chaenopsidae]) Studien über geschlechtsspezifische Unterschiede liefern Einblicke in Selektionsfaktoren, die auf Männchen und Weibchen wirken, besonders innerhalb Taxa, die hohe Level an Dimorphismus zeigen. Geschlechtsspezifische Unterschiede in Morphologie und Färbung (Melanophoren) für 66 der 89 Hechtschleimfischarten (Blenniiformes, Chaenopsidae) wurden aus Systematik-Literatur und durch Untersuchung von fixierten Proben zusammengetragen. Chaenopside umfassen grundsätzlich monomorphe Arten und solche, bei denen sich Männchen und Weibchen in bis zu 17 morphologischen und 14 Färbungsmerkmalen unterscheiden. Während sich die Geschlechter der meisten Arten in der Färbung unterscheiden (zumindest zur Paarungszeit), sind sie bei *Acanthemblemaria*, *Hemiemblemaria*, und *Lucayablennius* morphologisch ähnlich. Während andere Gattungen einen moderaten Level an Dimorphismus aufweisen, sind die Arten von *Coralliozetus*, *Cirriemblemaria*, und *Emblemaria* außerordentlich dimorph. Merkmalstabellen in Kombination mit phylogenetischen Daten weisen darauf hin, dass sich der extreme Dimorphismus in diesen Gattungen unabhängig entwickelt hat. Eine komplexe Evolutionsgeschichte wird durch die Untersuchung der Kieferlänge gestützt, wobei diese für ein oder beide Geschlechter zu- oder abnimmt, was entweder zu Dimorphismus oder Monomorphismus führt. Mehrere Merkmale, die im Zusammenhang mit der Schutzraum-Verteidigung stehen, sind in Arten, in denen beide Geschlechter Schutzräume bewohnen, monomorph, aber dimorph, wo nur Männer Schutzräume besetzen. Andere dimorphe Merkmale erhöhen die Auffälligkeit männlicher Werbung und aggressiver Zurschaustellung. Translated to German by S. Kruppert (skrupp@uw.edu)

Evolución del dimorfismo sexual en trambollos tubícolas (Teleostei: Chaenopsidae) (Evolution of sexual dimorphism in tube blennies [Teleostei: Chaenopsidae]) El estudio de las diferencias sexuales proporciona información sobre los factores selectivos que operan en hembras y machos especialmente para clados que muestran niveles variados de dimorfismo. Este estudio se enfocó en las diferencias sexuales en morfología y coloración de 66 especies de blenidos (Blenniiformes, Chaenopsidae). Para lo anterior se analizaron datos provenientes de literatura existente y el análisis de especímenes conservados. Los trambollos tubícolas incluyen especies esencialmente monomórficas y aquellas en las que los machos y las hembras difieren en 17 características morfológicas y 14 de coloración. Si bien los sexos de la mayoría de las especies difieren en la coloración (al menos en el momento de la reproducción), son morfológicamente similares en *Acanthemblemaria*, *Hemiemblemaria*, y *Lucayablennius*. Mientras que otros géneros exhiben un nivel intermedio de dimorfismo, las especies de *Coralliozetus*, *Cirriemblemaria*, y *Emblemaria* poseen un elevado dimorfismo sexual. La hipótesis filogenética indica que este nivel extremo de dimorfismo evolucionó independientemente en estos géneros. Al analizar la longitud de la mandíbula con aumentos y disminuciones en uno o ambos sexos que conducen a dimorfismo o monomorfismo se infiere una compleja historia evolutiva. Varias características relacionadas con la defensa del refugio son monomorfas en especies donde ambos sexos habitan en refugios, pero dimorfa donde solo los machos ocupan refugios. Otras características dimórficas aumentan la visibilidad del cortejo masculino y las exhibiciones agresivas. Translated to Spanish by S. Hinojosa (hinojosa.silvia@gmail.com)

Evolução do dimorfismo sexual em blennies tubulares (Teleostei: Chaenopsidae) (Evolution of sexual dimorphism in tube blennies [Teleostei: Chaenopsidae]) Estudo das diferenças sexuais provém novas idéias sobre fatores seletivos operando em machos e fêmeas, especialmente em clados que exibem níveis variados de dimorfismo. Diferenças sexuais na morfologia e coloração (melanofóros) foram compilados de 66 das 89 espécies de blennies tubulares (Blenniiformes, Chaenopsidae), examinando a literatura em sistemática e espécimes preservados. Chaenopídeos incluem espécies essencialmente monomórficas, além daquelas em que machos e fêmeas diferem em até 17 características morfológicas e 14 de coloração. Embora os sexos da maioria das espécies diferem em coloração (pelo menos no período de reprodução), eles são morfologicamente similares em *Acanthemblemaria*, *Hemiemblemaria*, e *Lucayablennius*. Enquanto outros gêneros exibem níveis intermediários de dimorfismo, espécies de *Coralliozetus*, *Cirriemblemaria*, e *Emblemaria* são dramaticamente dimórficas. Mapeamento de caracteres em uma hipótese filogenética indica que esses níveis extremos de dimorfismo surgiram independentemente nesses gêneros. Uma complexa história de evolução implica o envolvimento do comprimento das mandíbulas, com aumentos e reduções em um ou ambos sexos, levando ao dimorfismo ou monomorfismo. Diversas características relacionadas a defesa das tocas são monomórficas nas espécies em que ambos os sexos habitam tocas; mas nas espécies dimórficas, somente machos ocupam tocas. Outras características dimórficas aumentam a conspicuidade do cortejo masculino e demonstrações de agressividade. Translated to Portuguese by D. Vaz (dbistonvaz@vims.edu)

## Introduction

Sexual dimorphism has held a special fascination for evolutionary biologists since Darwin (1871) explored its significance. Sexually dimorphic features are among the most dramatic morphological attributes known, many of which appear at odds with the survival of individuals expressing them. The focus on sexual dimorphism has been particularly important in deciphering the disparate selective pressures on males and females within species (Hedrick and Temles 1989; Shine 1989; Berns 2013) and the literature on the evolution of sexual dimorphism is replete with studies documenting an advantage for the sex (usually males) bearing the unusual feature (Andersson 1984) or the size advantage afforded the larger of the two sexes (Badyaev 2002; Blanckenhorn 2005). While many studies have focused on single characters and often lack of a phylogenetic perspective (Wiens 2001; Badyaev and Hill 2003), an increasing number of studies, especially on birds, have interpreted the evolution of multiple dimorphic features within in a strict phylogenetic context (e.g., Berv and Prum 2014; Price and Eaton 2014; Gluckman and Munday 2016). These studies often reveal a complex pattern of evolution including divergence in females as well as in males, and the loss of sexual dimorphism (Wiens 2001; Gluckman 2014; Kraaijeveld 2014; Price 2015).

The current study was undertaken to quantify the degree of sexual dimorphism in the species of a family of fishes and to explore the pattern of evolution of these features within a phylogenetic framework. Teleost fishes include some of the most dimorphic of all species of vertebrates. Among these are the chaenopsid blennies, a group of blenniiform fishes that are particularly interesting for the range of sexual dimorphism expressed in a relatively small clade (Hastings and Springer 1994, 2009). This paper documents the pattern of sexual dimorphism in species of chaenopsids and explores its evolutionary history within this group.

**Fig. 1:**
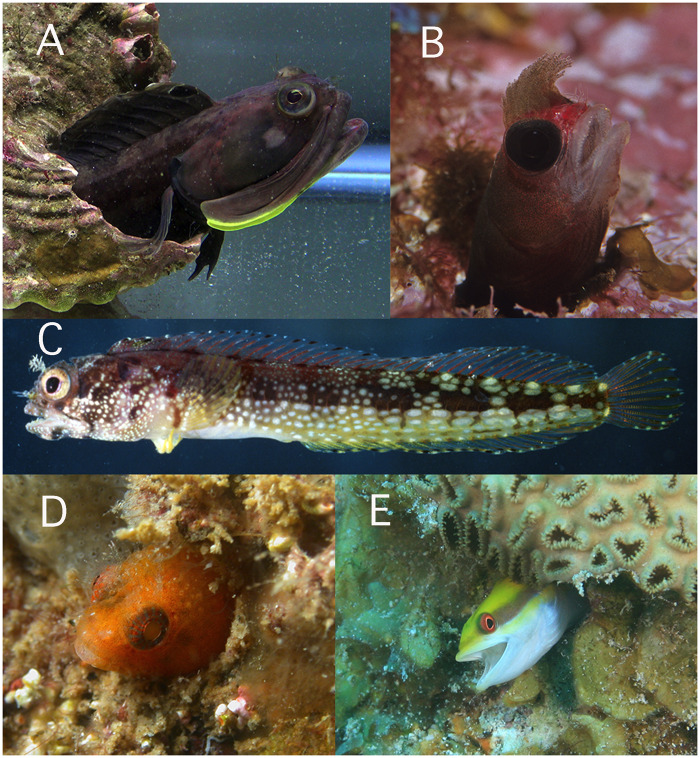
Relatively monomorphic chaenopsids. **A**) *Neoclinus blanchardi* male (Dimorphism score: M, morphology=2; C, coloration=0); **B**) *Mccoskerichthys sandae* (Dimorphism score: M = 1, C = 0); **C**) *Acanthemblemaria exilispinus* (Dimorphism score: M = 0, C = 0); **D**) *Protemblemaria bicirris* (Dimorphism score: M = 1, C = 4); **E**) *Hemiemblemaria simulus* (Dimorphism score: M = 0, C = 0)*.* Photo credits: A) by K. Bondy; B) by C. Bryce; C) by G. R. Allen; D) by A. Hermosillo; E) by K. Bryant [B) and C) from Shorefishes of the Eastern Pacific; D) and E) from Shorefishes of the Greater Caribbean].

**Fig. 2:**
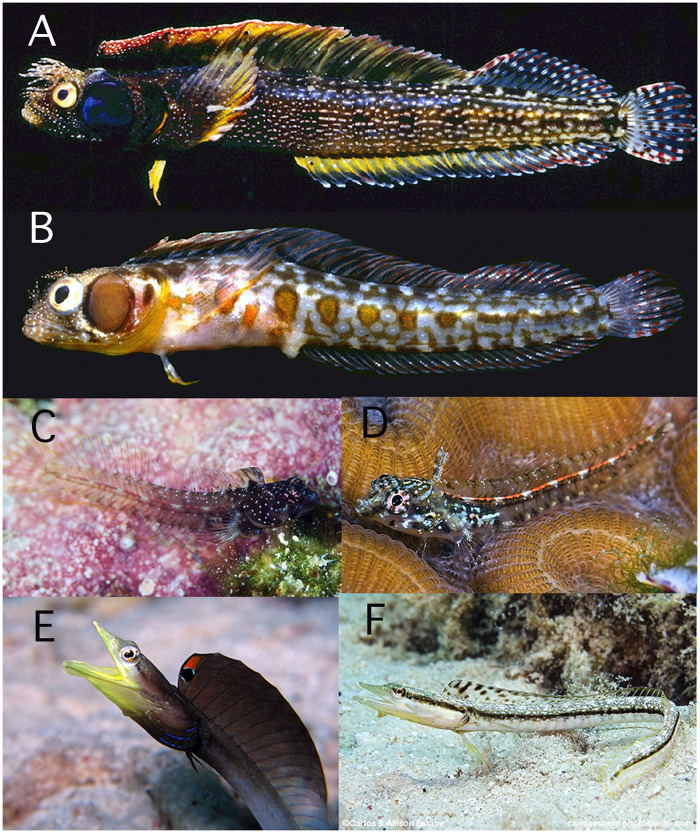
Moderately dimorphic chaenopsids. **A**) *Acanthemblemaria crockeri* male, and **B**) female (Dimorphism score: M = 0; C = 7); **C**) *Emblemariopsis diaphana* male, and **D**) female (Dimorphism score: M = 1, C = 10); **E**) *Chaenopsis limbaughi* male, and **F**) female (Dimorphism score: M = 3, C = 10). Photo credits: A) and B) by G.R. Allen (from Shorefishes of the Eastern Pacific); C), D), and F) by C. and E. Estape; E) by E. Muller (from Shorefishes of the Greater Caribbean).

**Fig. 3:**
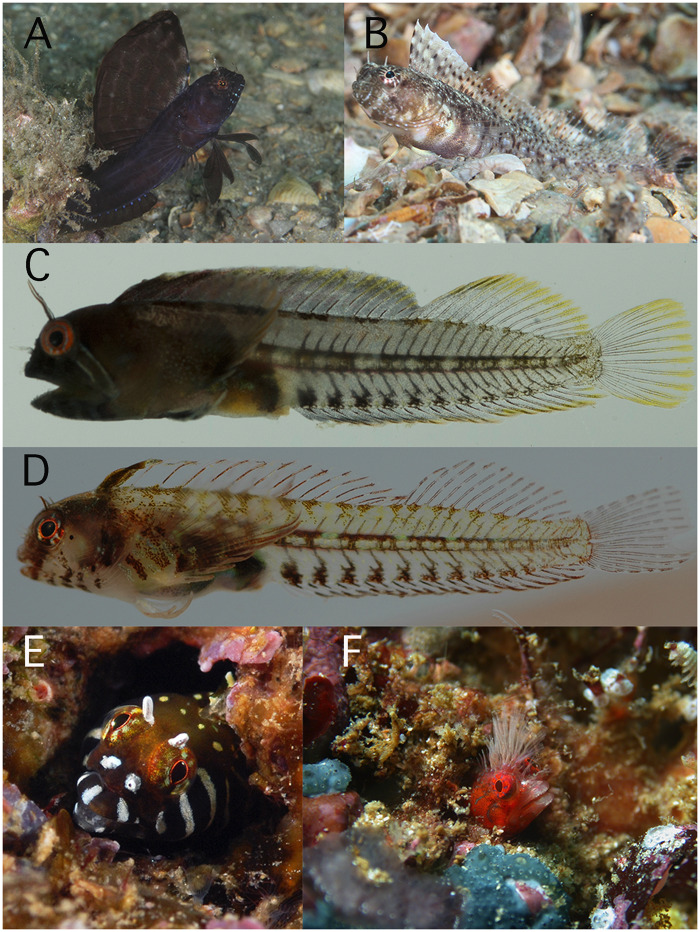
Highly dimorphic chaenopsids: **A**) *Emblemaria pandionis* male, and **B**) female (Dimorphism score: M = 5, C = 12); **C**) *Coralliozetus cardonae* male, and **D**) female (Dimorphism score: M = 17, C = 11); **E**) *Coralliozetus boehlkei* male (Dimorphism score: M = 17, C = 12); **F**) *Cirriemblemaria. lucasana* male (Dimorphism score: M = 13, C = 7). Photo credits: A) and B) by K. Bryant; C) and D) by J. Van Tassel and D. R. Robertson; E) by Kevin Lee; F) by A. Hermosillo (A–D from Shorefishes of the Greater Caribbean, E–F from Shorefishes of the Eastern Pacific).

The Chaenopsidae (*sensu*Lin and Hastings, 2011) comprises 89 known species including essentially monomorphic species (Fig. 1), a variety of somewhat dimorphic species (Fig. 2), as well as several strikingly dimorphic ones (Stephens 1963, 1970; Hastings 1991a, 2002). Three genera, *Coralliozetus* with 6 species, *Emblemaria* with 16 species, and the monotypic *Cirriemblemaria* are known to be especially dimorphic (Fig. 3). Chaenopsids are largely restricted to coastal waters of the Neotropics including the tropical eastern Pacific and tropical western Atlantic (Hastings 2009). Their common name of tube blennies reflects their typical occurrence in and dependency on vacant tests of invertebrates such as barnacles, mollusk shells, and polychaete tubes. Resident chaenopsids typically sit in a shelter with some or all of the head exposed. From this vantage point they forage on passing or nearby food items (typically small crustaceans; Kotrschal and Thomson 1986) and display to conspecifics, but rapidly retreat inside the shelter when startled. Thus, these shelters serve as refuges from predation (Hastings 1991b) and have been demonstrated to limit local population density (Buchheim and Hixon 1992; Hastings and Galland 2010), and are the focus of both intraspecific and interspecific competition (Clarke 1996; Clarke and Tyler 2003). In addition, shelters serve as egg-deposition sites where eggs are deposited by females and fertilized and guarded by resident males. Females prefer to mate with large males and males defending high quality (unfouled) shelters (Hastings 1988a, 1988b). Shelters of sufficiently high quality are therefore a necessary resource for male reproductive success and as such are the focus of intense male–male competition. Resident males court females from the entrance of their shelter, most often by partially extending from the shelter, erecting the dorsal fin (Fig. 3A), and rapidly retreating backward into the shelter. Occasionally courting males extend completely out of the shelter and some individuals periodically or regularly forage outside of shelters (Hastings 2002).

## Materials and methods

### Dimorphic characters

The systematic literature on chaenopsids was surveyed for descriptions of sexual dimorphism of all currently recognized species. This task was facilitated by the thorough and relatively consistent coverage of many species of chaenopsids by Stephens (1963, 1970). Additional references consulted for each of the 11 genera are as follows. *Acanthemblemaria*: Stephens et al. (1966), Smith-Vaniz and Palacio (1974), Acero (1984a), Rosenblatt and McCosker (1988), Johnson and Brothers (1989), Hastings (1990), Hastings and Robertson (1999); *Chaenopsis:*Böhlke (1957a), Robins and Randall (1965), Hastings and Shipp (1981); *Cirriemblemaria*: Hastings (1997); *Coralliozetus*: Stephens et al. (1966), Acero (1987), Hastings (1991a, 1997, 2002); *Ekemblemaria*: Acero (1984a), Hastings (1992a); *Emblemaria*: Johnson and Greenfield (1976), Acero (1984b), Williams (2002); *Emblemariopsis*: Cervigón (1999), Greenfield (1975), Greenfield and Johnson (1981), Tyler and Tyler (1997, 1999); *Hemiemblemaria*: Longley and Hildebrand (1941), Böhlke (1957a), Böhlke and Chaplin (1968); *Lucayablennius:* Böhlke (1957), Böhlke and Chaplin (1968), Greenfield (1972); *Mccoskerichthys*: Rosenblatt and Stephens (1978); *Neoclinus*: Hubbs (1953); *Protemblemaria*: Böhlke and Cervigon (1967) and Hastings (1997, 2001).

This literature compilation was revised and/or augmented by examination of preserved specimens in museum collections. All preserved specimens had been fixed in formalin and transferred to either 70% ethanol or 50% isopropanol. Primary specimens examined are listed in Supplementary Table S1, although additional specimens were examined for most species. These served to verify literature descriptions, to provide data on characters not uniformly covered in the literature (e.g., osteology, coloration), and to provide data on poorly known and incompletely described species. Adult males and females were examined for 66 of the 89 currently recognized species of chaenopsids (Supplementary Table S1). Species not included in this survey are listed in Supplementary Table S2.

Characters examined included morphological features (osteology, shape differences, soft anatomy, and fin shape and size) and coloration of the head, body, and fins (Table 1). Osteological characters were examined on cleared-and-stained specimens (Dingerkus and Uhler 1977) where available. Soft anatomy and coloration characters were examined on formalin-fixed and alcohol-preserved specimens (Supplementary Table S1). Morphometric characters included head shape, jaw length, lengths of the supraorbital and nasal cirri, and fin shapes and sizes. Head shape was subjectively scored as rounded (snout region steeply curving, snout length less than orbital diameter), pointed (snout less steeply curving, snout length approximately equal to orbital diameter), or elongate (snout longer than orbital diameter). Jaw length was scored as its posterior extent relative to other features of the head including the posterior margin of the orbit, the preopercle, or the opercle. Several other morphometric characters reported to be sexually dimorphic in some chaenopsids were not included in this survey because of lack of data for most species. These included length of the abdomen which is reportedly larger in females of *Neoclinus uninotatus*, *N. blanchardi*, *N. okazaki*, and *Coralliozetus angelicus* (Hubbs 1953; Fukao 1990; Hastings 1991a), head length which is reportedly greater in males of *Cirriemblemaria lucasana* (Stephens 1963), interorbital width and pelvic-fin length which are reportedly greater in male *Coralliozetus micropes* (Stephens 1963), and length of spines on the head which is reportedly greater in males of *Acanthemblemaria crockeri* (Stephens 1963). Two meristic characters, number of palatine teeth and number of precaudal vertebrae, reportedly dimorphic in *C.angelicus* (Hastings 1991a) were also not included. Finally, size dimorphism reported for several species of chaenopsids (e.g., Hastings 1991a; Hastings and Robertson 1999) will be considered in a separate study.

**Table 1:** Sexually dimorphic characters of chaenopsids, including general character condition in monomorphic species, observed sexual differences, and sexually dimorphic taxa

Character	General condition in monomorphic species	Observed sexual differences	Dimorphic taxa
M-I. Morphology: Osteology			
M-Ia. Infraorbital size	Thick or slender	More slender in females	Various species
M-Ib. Infraorbital number	Two or four	Three in females	*Coralliozetus* spp.
M-Ic. Infraorbital texture	Variable: smooth, pits, spines, or ridges	Smoother in females	Various species
M-Id. Nasal fusion	Fused or separate	Fused in males, separate in females	*Coralliozetus* spp.
M-Ie. Nasal texture	Variable: smooth, pits, spines, ridges or knobs	Smoother in females	Various species
M-II. Morphology: Morphometrics			
M-IIa. Head shape	Variable: rounded, pointed, or elongate	More pointed in females	Various species
M-IIb. Jaw length	Variable: from mid-orbit to past operculum	Shorter in females	Various species
M-IIc. Supraorbital cirrus length	Variable: absent, or < half orbital diameter to ≫ orbital diameter	Shorter in females	Various species
M-IId. Nasal cirrus length	Variable: absent, tiny, short, or long	Shorter in females	Various species
M-III. Morphology: Soft Anatomy			
M-IIIa. Lip shape	Flat	Protruding in females	*Coralliozetus* spp.
M-IIIb. Dewlap on chin	Absent or present	Larger in males	Two *Chaenopsis* spp.
M-IIIc. Snout flaps	Absent	Present in males	*C. lucasana*
M-IIId. Nape papillae	Absent	Present in males	*C. lucasana*
M-IIIe. Nape folds	Absent	Present in males	*Emblemaria* spp.
M-IIIf. Nape muscles	Present or absent	Absent in females	*Coralliozetus* spp.
			Some *Emblemaria* spp.
M-IIIg. Dorsal sensory pores	Few to many	Fewer in males	*Coralliozetus* spp.
M-IV. Morphology: Fins			
M-IVa. Dorsal fin shape	Variable	Variable	Various species
M-IVb. Dorsal fin size	Variable	Larger in males	Various species
M-IVc. Dorsal fin flap	Variable: absent, thin, moderate, or flaglike	Present in males, absent in females	*Emblemaria* spp.
			*Emblemariopsis* spp.
M-IVd. Dorsal fin notch	Absent, slight, or deep	Deeper in females	Various species
M-IVe. Pectoral fin shape	Rounded or pointed	More pointed in females	Various species
M-IVf. Pelvic fin rays	Incised or not (membrane to ray tips)	Not incised in males, incised in females	Some *Emblemaria* spp.
C-I. Coloration: Head			
C-Ia. Head	Variable	Darker in males	Most species
C-Ib. Lower jaw	Variable	Uniform, darker in males; banded in	Various species
		females	
C-Ic. Branchiostegals	Variable	Darker in males	Most species
C-II. Coloration: Body			
C-IIa. Lateral body	Variable	Darker in males	Various species
C-IIb. Abdomen	Variable	Darker in males	Various species
C-IIc. Anterior of pelvic fins	Variable	Clear areas in females	*Ekemblemaria* spp.
C-III. Coloration: Fins			
C-IIIa. Dorsal fin, anterior	Variable	Darker in males	Most species
C-IIIb. Dorsal fin, spot(s)	Variable	Present or absent in either sex	Various species
C-IIIc. Dorsal fin, posterior	Variable	Darker in males	Various species
C-IIId. Dorsal fin “windows”	Absent	Clear spots present in females	*Ekemblemaria* spp.
C-IIIe. Caudal fin	Variable	Darker in males	Various species
C-IIIf. Anal fin	Variable	Darker in males	Most species
C-IIIg. Posterior median fins	Variable	More clear area in females	*Ekemblemaria* spp.
C-IIIh. Pectoral fin base	Variable	Darker in males	Various species
C-IIIi. Pectoral fin	Variable	Darker in males	Various species
C-IIIj. Pectoral fin bands	Absent	Present in females	*C. micropes*
C-IIIk. Pelvic fin	Variable	Darker in males	Various species

Because life colors are unknown for most species, this study focused on color features retained in formalin-fixed and alcohol-preserved specimens, i.e., the distribution and density of melanophores. Melanophore distribution and density is often extremely variable in chaenopsids, both within and across the sexes of conspecifics. For most species, maximum sexual color differences occur during the breeding season when the density, expansion, and thus prominence of melanophores (and bright colors in many species) increases in males. An attempt was made to examine collections that included males with a well-developed genital papilla and females with a highly fimbriate genital area (see Böhlke 1957b, Fig. 2), and collections in which the greatest differences in coloration of the sexes were observed. Consequently, color differences reported herein reflect the greatest difference between the sexes as far as detectable given available specimens. Many chaenopsids (e.g., all species of *Coralliozetus*) exhibit dramatic dimorphism in bright colors during breeding and also retain a significant level of color dimorphism in the non-breeding season. In others, sexual differences in melanophore concentration are maintained throughout the breeding season, with the sexes reverting to similar coloration in the non-breeding season. In a few chaenopsids, males exhibit unique color patterns (e.g., expanded melanophores) only while courting, quickly reverting to a coloration similar to that of females when not courting (e.g., *Acanthemblemaria exilispinus*, personal observation). Such features could not be detected in this study, and no attempt was made to quantify these ephemeral color differences or to distinguish between permanent and temporary sexual dichromatism.

Degree of color dimorphism was assessed by scoring the pattern and/or relative density of melanophores of males and females on the head, body, and fins (Table 1). Three characters were scored on the head (overall head coloration and that on the lower jaw and branchiostegal membranes), 3 characters were scored on the body (lateral aspect, abdomen, and region anterior to the pectoral fin), and 11 were scored on the fins (Table 1). These included the location of anterior dorsal-fin spots, other pigment on the anterior (spinous) dorsal fin, posterior (segmented-ray portion) dorsal fin, caudal fin, anal fin, pectoral fin, and pelvic fin. In some cases these were scored as both the pattern of melanophore distribution and the density of melanophores when one or more of the study species exhibited a sex-related difference in that particular region.

These characters are not considered to be completely independent (see Emerson and Hastings 1998) and many are not suitable as typical phylogenetic characters (and consequently are termed “scores” instead of “states”). Resolution of phylogenetic relationships is not the intent of this survey, and some character scores reflect an arbitrarily divided, graded degree of divergence of the sexes. For example, courting males of many species become increasing dark via an increase in the density and expansion of melanophores. This is often expressed on the branchiostegal membranes and head, but may extend across the entire head, body, and fins in some species.

Character scores for males and females of all 66 included species were recorded for all 39 characters found to be sexually dimorphic in any species (Supplementary Table S3). These data are summarized in Table 2 that record the number of characters within each category differing in males and females for each species, as well as the total numbers of dimorphic morphological, coloration, and all characters for each species. Summary statistics for the 11 currently recognized genera are given in Table 3.

**Table 2:** Number of sexually dimorphic characters for morphology, coloration, and total for each species

	Morphology					Coloration				Total
	M-I	M-II	M-III	M-IV	M-Total	C-I	C-II	C-III	C-Total	
Number of characters in category	5	4	7	6	22	3	3	11	17	39
*Acanthemblemaria*										
* aspera*	0	0	0	0	0	2	2	6	10	10
* atrata*	0	0	0	0	0	1	0	0	1	1
* balanorum*	0	0	0	0	0	1	0	0	1	1
* betinensis*	0	0	0	0	0	3	1	2	6	6
* castroi*	0	0	0	0	0	2	1	3	6	6
* chaplini*	0	0	0	0	0	3	2	5	10	10
* crockeri*	0	0	0	0	0	2	2	3	7	7
* exilispinus*	0	0	0	0	0	0	0	0	0	0
* greenfieldi*	0	0	0	0	0	1	1	0	2	2
* hancocki*	0	0	0	0	0	3	1	5	9	9
* harpeza*	0	0	0	0	0	1	1	1	3	3
* hastingsi*	0	0	0	0	0	3	2	5	10	10
* macrospilus*	0	0	0	0	0	3	2	5	10	10
* mangognatha*	0	0	0	0	0	2	0	1	3	3
* maria*	0	0	0	0	0	0	0	0	0	0
* medusae*	0	0	0	0	0	3	1	3	7	7
* paula*	0	2	0	0	2	1	1	0	2	4
* rivasi*	0	0	0	0	0	2	2	6	10	10
* spinosa*	0	0	0	0	0	2	1	3	6	6
* stephensi*	0	0	0	0	0	2	2	6	10	10
*Chaenopsis*										
* alepidota*	0	0	0	2	2	3	2	5	10	12
* coheni*	0	0	0	2	2	3	2	6	11	13
* deltarrhis*	0	0	0	2	2	3	1	6	10	12
* limbaughi*	0	0	1	2	3	3	2	5	10	13
* ocellata*	0	0	0	2	2	3	2	3	8	10
* resh*	0	0	1	2	3	3	2	7	12	15
* roseola*	0	0	0	0	0	2	0	3	5	5
* schmitti*	0	0	0	2	2	3	0	6	9	11
* new species*	0	0	0	2	2	2	0	3	5	7
*Cirriemblemaria*										
* lucasana*	3	3	3	4	13	1	0	6	7	20
*Coralliozetus*										
* angelicus*	5	4	3	5	17	3	2	8	13	30
* boehlkei*	5	4	3	5	17	3	2	7	12	29
* cardonae*	5	4	3	5	17	3	2	6	11	28
* micropes*	5	4	3	5	17	3	2	9	14	31
* rosenblatti*	5	3	3	5	16	3	2	8	13	29
* springeri*	5	4	3	5	17	3	2	6	11	28
*Ekemblemaria*										
* myersi*	0	1	0	0	1	1	2	3	6	7
* nigra*	0	1	0	0	1	1	1	3	5	6
*Emblemaria*										
* atlantica*	3	4	1	4	12	3	2	6	11	23
* caldwelli*	0	1	0	4	5	3	2	6	11	16
* caycedoi*	0	1	1	2	4	3	1	5	9	13
* diphyodontis*	3	3	1	5	12	3	2	7	12	24
* hudsoni*	3	4	1	5	13	3	2	7	12	25
* hyltoni*	0	2	0	1	3	3	2	6	11	14
* hypacanthus*	3	4	1	6	14	3	2	7	12	26
* nivipes*	0	0	0	4	4	3	2	8	13	17
* pandionis*	0	1	0	4	5	3	2	7	12	17
* piratica*	3	3	1	5	12	3	2	7	12	24
* piratula*	2	2	0	5	9	3	2	7	12	21
* walkeri*	3	3	1	5	12	3	0	4	7	19
*Emblemariopsis*										
* bahamensis*	3	2	1	0	6	3	2	6	11	17
* diaphana*	1	1	0	0	2	3	2	5	10	12
* leptocirris*	1	2	0	0	3	3	2	5	10	13
* occidentalis*	2	2	1	0	5	3	2	5	10	15
* pricei*	0	0	0	0	0	3	1	5	9	9
* randalli*	1	1	1	0	3	1	1	5	7	10
* signifera*	2	1	1	1	5	3	2	7	12	17
*Hemiemblemaria*										
* simulus*	0	0	0	0	0	0	0	0	0	0
*Lucayablennius*										
* zingaro*	0	0	0	0	0	0	1	2	3	3
*Mccoskerichthys*										
* sandae*	0	1	0	0	1	0	0	0	0	1
*Neoclinus*										
* blanchardi*	0	1	0	1	2	0	0	0	0	2
* stephensae*	0	1	0	0	1	2	1	6	9	10
* uninotatus*	0	2	0	1	3	0	0	0	0	3
*Protemblemaria*										
* bicirris*	0	0	0	0	0	2	0	0	2	2
* perla*	0	0	0	0	0	1	0	4	5	5
* punctata*	0	0	0	1	1	2	0	2	4	5

M-I=osteology; M-II=morphometrics; M-III=soft anatomy; M-IV=fins; M-Total=all morphological characters; C-I=head coloration; C-II=body coloration; C-III=fin coloration; C-Total=all coloration characters; Total=combined morphology and coloration characters (Table 1).

**Table 3:** Summary of sexually dimorphic features for genera of chaenopsids

	Morphology (22)	Coloration (17)	Total (39)
*Acanthemblemaria* (20/21)	0.1 (0.4) 0–2	5.6 (3.8) 0–10	5.7 (3.7) 0–10
*Chaenopsis* (9/11)	2.0 (0.9) 0–3	8.9 (2.5) 5–12	10.9 (3.1) 5–15
*Cirriemblemaria* (1/1)	13 (−) 13	7 (−) 7	20 (−) 20
*Coralliozetus* (6/6)	16.8 (0.4) 16–17	12.3 (1.2) 11–14	29.2 (1.2) 28–31
*Ekemblemaria* (2/3)	1.0 (0) 1	5.5 (0.7) 5–6	6.5 (0.7) 6–7
*Emblemaria* (12/16)	8.7 (4.2) 3–14	11.1 (1.6) 7–13	19.9 (4.5) 13–26
*Emblemariopsis* (7/14)	3.4 (2.1) 0–6	9.9 (1.6) 7–12	13.3 (3.2) 9–17
*Hemiemblemaria* (1/1)	0 (−) 0	0 (−) 0	0 (−) 0
*Lucayablennius* (1/1)	0 (−) 0	3 (−) 3	3 (−) 3
*Mccoskerichthys* (1/1)	1 (−) 1	0 (−) 0	1 (−) 1
*Neoclinus* (3/11)	2.0 (1.0) 1–3	3.0 (5.2) 0–9	5.0 (4.4) 2–10
*Protemblemaria* (3/3)	0.3 (0.6) 0–1	3.7 (1.5) 2–5	4.0 (1.7) 2–5
All genera (66/89)	4.1 (5.6) 0–17	7.7 (4.1) 0–14	11.9 (8.6) 0–31

Numbers in parentheses in heading are numbers of dimorphic characters in that category, those after genera are number of species included in survey/total number of species in genus. Means (standard deviation) ranges are for characters listed in Table 1 and scored in Table 2.

### Phylogenetic relationships and character mapping

The pattern of sexual dimorphism in chaenopsids was analyzed on a composite phylogenetic hypothesis assembled from several recent studies. The phylogenetic analysis of Lin and Hastings (2011) included 35 species and was based on both molecular data (829 parsimony informative characters) and morphological data (145 parsimony informative morphological characters that included 19 sexually dimorphic characters). The most parsimonious tree reflected the signal in the molecular data, thus the morphological characters did not affect the final outcome. The monophyly of all currently recognized genera was supported by one or more morphological synapomorphies and confirmed with genetic data (Lin and Hastings 2011). The relationships of *Neoclinus* and its sister group, the monotypic *Mccoskerichthys*, to the Chaenopsidae are unresolved. Although considered closely related to, or members of, the Chaenopsidae (Stephens 1963; Rosenblatt and Stephens 1978; Hastings and Springer 1994; Lin and Hastings 2011), the relationships of this clade were not clearly resolved in a recent genetic analysis of blenniiform fishes. It emerged along with chaenopsids within a poorly resolved region of short branch lengths in the blenniiform tree (Lin and Hastings 2013). Both *Neoclinus* and *Mccoskerichthys* are hole-dwelling as are other chaenopsids, and are included in this study as the sister group of the Chaenopsinae (*sensu*Lin and Hastings 2011). Genetic data indicate that the enigmatic genus *Stathmonotus*, sometimes included in the Chaenopsidae (Hastings and Springer 1994), is more closely related to the paraclinin labrisomids (Lin and Hastings 2013) and is not included in this study.

**Fig. 4:**
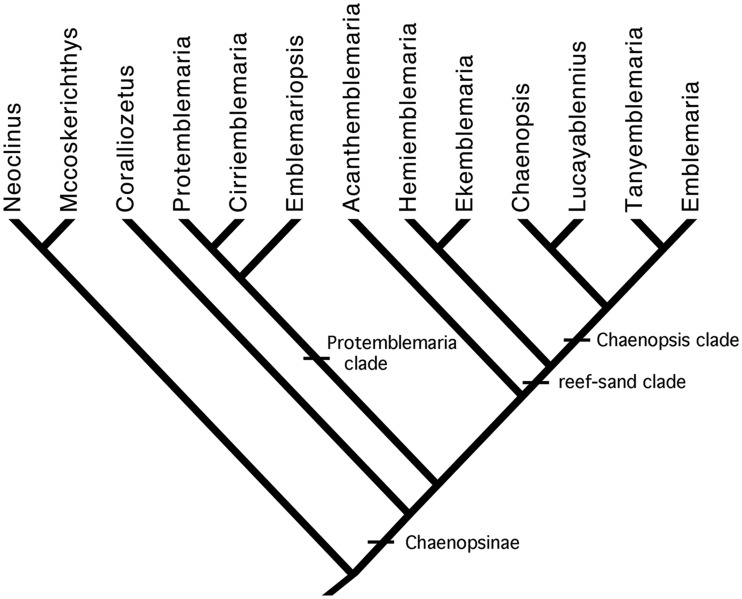
Phylogenetic relationships of chaenopsid genera with internal clades indicated.

Within the Chaenopsinae (Fig. 4) the distinctive genus *Coralliozetus* (Hastings 2002) with six species is sister to all others, followed by a clade with *Protemblemaria* (three species), *Cirriemblemaria* (one species), and the western Atlantic endemic genus *Emblemariopsis* (14 species). This lineage is sister to a large clade that includes *Acanthemblemaria* with 21 species, and a “reef-sand clade” (see Lin and Hastings 2011) that includes *Ekemblemaria* (three species; Hastings 1992a) and the monotypic *Hemiemblemaria*, plus the “*Chaenopsis* clade” (Hastings 1992b; Lin and Hastings 2011). The latter clade includes the monotypic genera *Lucayablennius*, and *Tanyemblemaria*, and two relatively speciose genera, *Chaenopsis* with 11 species, and *Emblemaria* with 16 species.

**Fig. 5:**
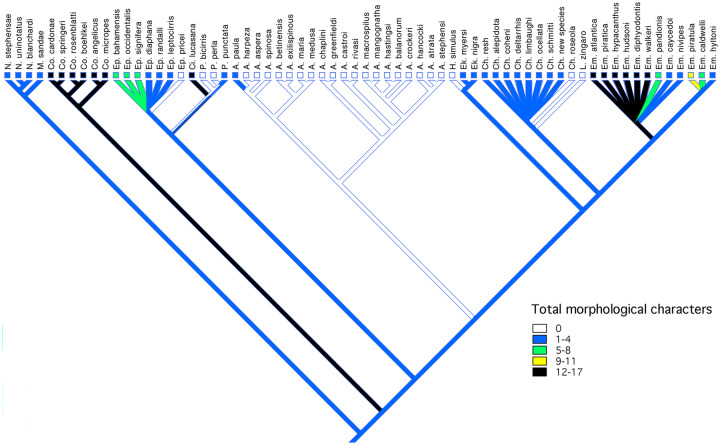
Character map of number of sexually dimorphic morphological features on the composite phylogeny.

Species-level relationships of chaenopsids have been hypothesized for some but not all genera. Relationships of the six species of *Coralliozetus* are incompletely resolved due to conflicts in characters, however, the “total evidence” tree based on both molecular and morphological data (Lin and Hastings 2011) is followed here (Fig. 5). Species-level relationships were hypothesized for *Protemblemaria* by Hastings (2001) using morphological characters (none sexually dimorphic), and for *Acanthemblemaria* most recently using genetic data by Eytan et al. (2012) but also using morphological data (60 characters, one sexually dimorphic) by Hastings (1990), Almany and Baldwin (1996), and Hastings and Robertson (1999). Species-level relationships within *Emblemariopsis*, *Chaenopsis*, and *Emblemaria* have not been studied in detail and consequently are for the most part represented in this study as polytomies (Fig. 5). Stephens’ (1963) hypothesis of relationships of species of *Emblemaria* known at that time was largely based on similarity. However, a four-species clade within *Emblemaria*, the “*caldwelli* species group,” with a uniquely reduced third pelvic-fin ray (Johnson and Greenfield 1976; Williams 2002) is recognized here.

Using results from these separate studies, a composite phylogenetic hypothesis (Fig. 5) was constructed and used to trace the evolution of sexual dimorphism by plotting the number of sexual dimorphic morphological, coloration, and total characters in each species as follows. Morphological characters were arbitrarily scored as: 0 = no dimorphic characters; 1 = 1–4 characters; 2 = 5–8; 3 = 9–11; and 4 = 12–17. Coloration characters were scored as: 0 = none; 1 = 1–4; 2 = 5–8; 3 = 9–11; and 4 = 12–14. Total characters were scored as: 0 = none; 1 = 1–6; 2 = 7–13; 3 = 14–19; and 4 = 20–31. The presence of unresolved polytomies in the phylogenetic hypothesis for the genera *Emblemariopsis*, *Emblemaria*, and *Chaenopsis* precluded stochastic character mapping. Consequently these characters were considered unordered and the most parsimonious resolutions were mapped on the phylogenetic hypothesis using Mesquite 3.31 (Maddison and Maddison 2017). Similarly, the posterior extend of the jaws relative to the orbit and preopercular margin of males and females, and presence/absence of sexual dimorphism in this feature were similarly mapped using Mesquite 3.31.

## Results

This study revealed striking variation in the degree of sexual dimorphism across the Chaenopsidae. The lineage includes essentially monomorphic species as well highly dimorphic species that exhibit differences in a variety of phenotypic characters ranging from osteological features to both transient and permanent color differences (Table 1). Among the 66 species included in this survey, the total number of sexually dimorphic characters ranged from 0 to 17 for morphological characters, 0 to 14 for coloration characters, and 0 to 31 for all characters (Table 2). These numbers represent a minimum number of sexually dimorphic characters as additional dimorphic characters would be revealed through morphometric analyses (e.g., abdomen length is reportedly dimorphic in some species), examination of additional specimens collected near the time of breeding (e.g., transient color differences), and if more specimens of poorly known species were available.

### Types of sexually dimorphic characters

Several chaenopsids are sexually dimorphic in osteological features in the head area. In these, the general pattern is for males to have more heavily ossified bones than females (Table 1). For example, the infraorbitals are thicker and larger in males of *Coralliozetus* (see Hastings 1991a, Fig. 3b), *Cirriemblemaria*, and *Emblemaria*. In most relatively monomorphic species, the infraorbitals are thick and heavily ossified in both sexes (e.g., species of *Acanthemblemaria*), while in a few they are slender in both sexes (e.g., *Lucayablennius zingaro*). Associated with a decrease in robustness of the head, pedomorphic females of all species of *Coralliozetus* have three infraorbital elements instead of two as in male *Coralliozetus* and both sexes of other chaenopsins (Hastings 1991a, Fig. 3a, 2002). Both sexes of *Neoclinus* and *Mccoskerichthys* have four relatively thick infraorbitals.

Relative jaw length differs greatly among chaenopsids and across the sexes of many species (Fig. 8). In males of most species, the posterior tip of the maxilla extends beyond the level of the posterior margin of the orbit, and in some cases extends posteriorly well past the posterior margin of the preopercle, and well past the posterior margin of the opercle in *Neoclinus blanchardi*. In females of many species, the jaws are similarly long (not dimorphic), while in others, the jaws are shorter than those of males. The relative size of the jaws of both males and females has evolved frequently among chaenopsids (Fig. 8).

Several aspects of shape and soft anatomy, especially in the head region, differ between male and female chaenopsids (Table 1). In most species, males and females have similar head shapes, but in females of all species of *Coralliozetus*, and some species of *Emblemaria* and *Emblemariopsis*, females have more pointed heads than males. The relative lengths of the supraorbital cirri and, less frequently, the nasal cirri are dimorphic in several species and these cirri are consistently longer in males. A number of other exterior soft-tissues features, such as additional cirri or fleshy ridges on the head, have evolved sporadically in chaenopsids and in species in which such features are sexually dimorphic, they are consistently larger in males.

The fins of chaenopsids show an extraordinary degree of variation within and across species (Table 1), and it is here that some of the most striking sexually dimorphic features are evident. Chief among these is the size and shape of the anterior dorsal fin, which is typically low and more-or-less even in profile in most species. In several dimorphic species, the anterior dorsal fin is elevated in males, being sail-like in several including species of *Emblemaria* (Fig. 3A), *C.micropes*, and some species of *Chaenopsis* (Fig. 2E), and spike-like in a few (e.g., *Coralliozetus rosenblatti*, *Emblemariopsis signifera*). In these, the dorsal fin of females is lower than that of males (Figs. 2F, 3B). Differences between males and females in more posterior regions of the dorsal fin as well as the other fins are seen in some chaenopsids. The pectoral fins of most species are rounded in both sexes, and pointed (with central rays considerably longer than those above and below) in both sexes of a few. In a number of species the fins of males are rounded while those of females are pointed. The pelvic fins of both sexes of most chaenopsids, like those of most other blenniiforms, are deeply incised and the free rays serve as props for resting on the substrate. However in several species of *Emblemaria*, the pelvic fins of males have enlarged membranes between the segmented rays that extend the full length of the rays, while females have typical incised fins. In courting males these are often heavily pigmented (Fig. 3A) and especially conspicuous during displays (Smith et al. 1998).

Sex-based dichromatism is common and widespread in chaenopsids. In most cases, males have more dense melanophores than females, especially during the breeding season (Table 1). This includes the overall head region and the branchiostegal membrane in most species, but also extends posteriorly to the lateral body and fins in many (e.g., Fig. 3C,D). In a number of species the lower jaw coloration is dimorphic with a general pattern that females have bands on the lower jaw, while males have darker, more uniformly colored lower jaws (e.g., Fig. 3C,D). It is clear from Table 1 that sexual dichromatism is most prominent in features associated with the head and anterior portions of the body and fins. The anterior dorsal fin is an especially variable region in which the sexes of many chaenopsids differ in the size and shape of this fin as well as in overall coloration and the position of a spot or spots (e.g., Fig. 2E,F). Posteriorly, male and female chaenopsids more closely resemble one another in coloration as well as in morphology.

### Patterns of dimorphism in chaenopsids

Not surprisingly, sexual dimorphism is not uniformly distributed among chaenopsids. Rather, some lineages are relatively monomorphic while others are highly dimorphic. Members of the *Neoclinus* clade, sister to the Chaenopsinae, are relatively monomorphic with the sexes differing most prominently in the length of the jaw which is extraordinarily long in males of the large-bodied species (Hongjamrassilp et al. 2018) and in a few color characters. Males and females of the monotypic *Mccoskerichthys* differ in only one morphological character, jaw length, and no coloration differences are known, although breeding coloration for this species has not been described.

Within the Chaenopsinae, the six species of the genus *Coralliozetus* (Fig. 3C–E) exhibit the greatest degree of sexual dimorphism, with males and females differing in 17 morphological features and 11–14 coloration characters (Table 2). The “*Protemblemaria* clade” includes the relatively monomorphic genus *Protemblemaria*, the highly dimorphic monotypic genus *Cirriemblemaria* (Fig. 3F), and the variably dimorphic genus *Emblemariopsis* (Table 2). Most species in the genus *Acanthemblemaria* are relatively monomorphic, especially with regard to morphological features (Table 2). Their most striking sexual differences center around coloration. A few species, such as *A. crockeri* (Fig. 2A,B) and *A. aspera*, exhibit permanent differences in coloration, especially along the sides of the body in the former (Stephens 1963). More commonly, sexual differences in coloration result from increased development of melanophores in males at the time of breeding. These differences in breeding coloration are most conspicuous on the head, especially the branchiostegal region, and on the anterior dorsal fin, but are expressed throughout the body in some species. At least one species, *A. exilispinus* (Fig. 1C), exhibits no apparent sexual differences in coloration (or other features) although males take on a distinctive dark head coloration with lighter eyes while courting (personal observations). Within the “reef-sand clade,” species of the genus *Ekemblemaria* are relatively monomorphic with the exception of jaw length, supraorbital cirrus length, and coloration (Hastings 1992a). The two included species exhibit a number of unique sexual differences in coloration (Table 2), most of which are permanent rather than expressed at the time breeding. Sister to this genus is the relatively monomorphic Wrasse Blenny, *Hemiemblemaria simulus* (Fig. 1E). Members of the “*Chaenopsis* clade” exhibit a wide range in degree of sexual dimorphism, including relatively monomorphic species such as *L.zingaro* (Fig. 1D) and *Chaenopsis roseola*, moderately dimorphic species of *Chaenopsis* (Fig. 2D–F), and some highly dimorphic species in the genus *Emblemaria* (Fig. 3A,B). The latter genus exhibits the greatest range in number of dimorphic characters of any chaenopsid genus (Table 3). Members of the “*caldwelli* species group,” as well as *Emblemaria nivipes*, are relatively monomorphic (Table 2), while others are conspicuously dimorphic.

### Evolution of dimorphism

**Fig. 6:**
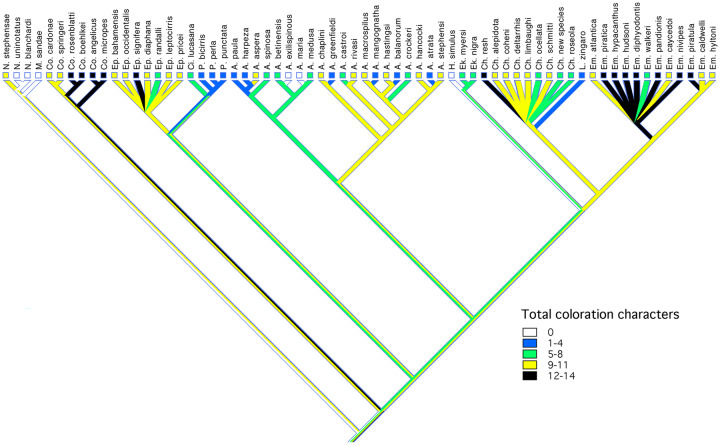
Character map of number of sexually dimorphic coloration features on the composite phylogeny.

**Fig. 7:**
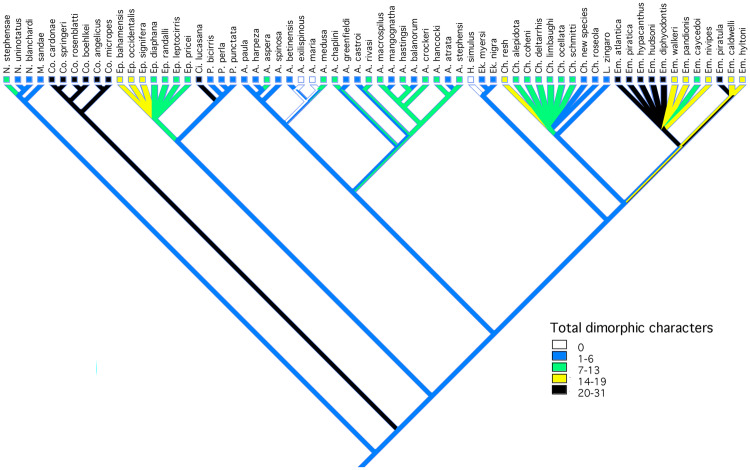
Character map of total number of sexually dimorphic features on the composite phylogeny.

Mapping the number of sexually dimorphic characters for species of chaenopsids on the composite phylogeny for the family (Figs. 5–7) indicates a complicated evolutionary history. For morphological characters (Fig. 5), the plesiomorphic condition within the family is relative monomorphism. In *Neoclinus* and *Mccoskerichthys*, the sexes differ primarily in jaw length with males having a longer jaw. Monomorphism in morphology is characteristic of many members of the Chaenopsinae, including the genera *Acanthemblemaria*, *Hemiemblemaria*, *Lucayablennius*, two species of *Protemblemaria*, and at least one species of *Chaenopsis*. A relatively high degree of morphological dimorphism (over 11 features) has evolved independently in *Coralliozetus*, *Cirriemblemaria*, and within *Emblemaria*. These include degree of ossification of selected bones of head, development of soft tissues including most notably the supraorbital cirri, as well as several differences in the shape and size of fins.

**Fig. 8:**
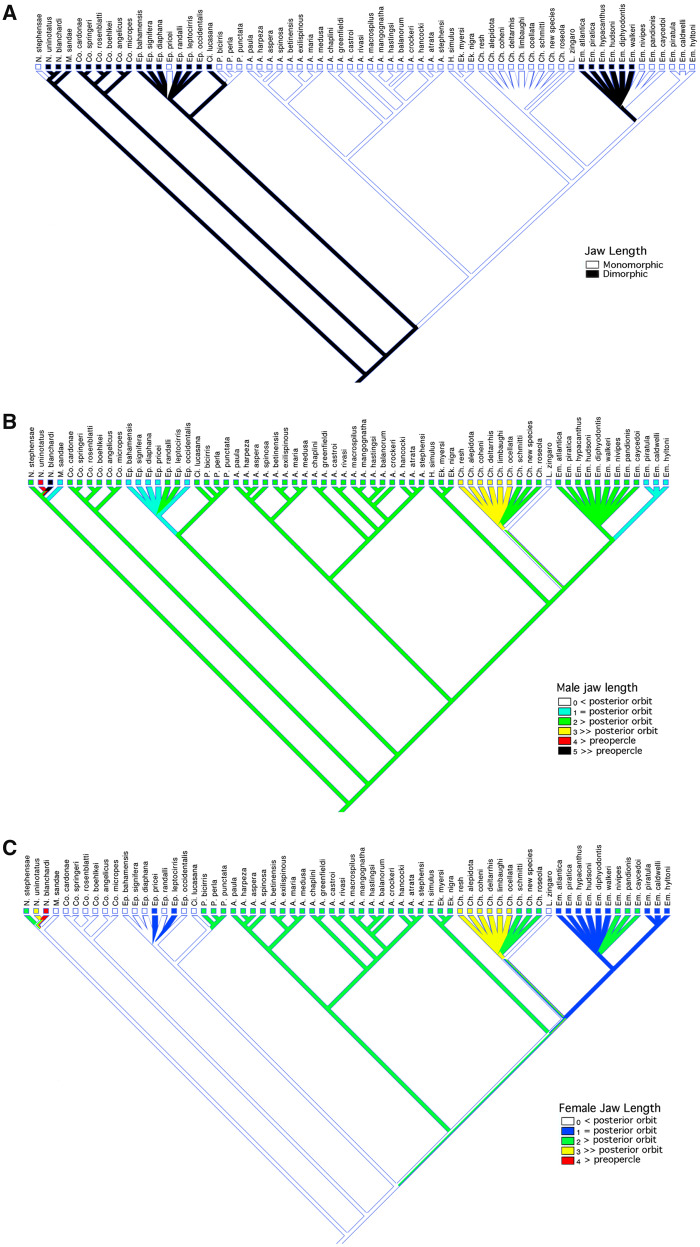
Character map for: **A**) dimorphism in relative jaw length; **B**) relative jaw length of males; and **C**) relative jaw length of females.

The complexity of evolution of sexual differences in chaenopsids is exemplified by the varied pattern of changes in the relative length of the jaws (Fig. 8). Although the lack of resolution of relationships within several lineages limits the ability to fully resolve evolution of this character, it is clear that its evolutionary history involves both increases and decreases in relative jaw size, with evolution in males alone, in females alone, and simultaneously in both sexes. The ancestral condition appears to be sexual dimorphism with the jaws of males extending past the posterior margin of the orbit and those of females falling short of the posterior orbital margin. Mapping presence/absence of dimorphism indicates that the sexes diverged at least four times assuming a minimum number of steps within polytomies (Fig. 8A). However, mapping relative jaw length in males and females separately indicates that this feature has changed at least seven times in males (Fig. 8B) and at least 10 times in females (Fig. 8C). Within the *Neoclinus* lineage, jaw dimorphism is present in *Mccoskerichthys*, and greatly exaggerated in two species of *Neoclinus* by elongation of the jaws in males, while monomorphism apparently evolved in *N. stephensae* via elongation of the jaws in females. Monomorphism evolved via elongation of the jaws of females in *Protemblemaria* and independently in *Acanthemblemaria* and possibly in the *Ekemblemaria/Hemiemblemaria* clade (character map equivocal, Fig. 8B). Monomorphism was maintained despite elongation of the jaws of both sexes in most species of *Chaenopsis*, but via reduction of jaw length in both sexes of *L.zingaro*. Within *Emblemaria*, dimorphism evolved via reduction in jaw length in females. Similar complex patterns of evolution are seen in other morphological characters included in this survey.

Sexual differences in coloration (melanophore distribution) are more widespread in chaenopsids including in most otherwise monomorphic species (Fig. 6). In the genus *Acanthemblemaria*, the sexes differ almost exclusively in coloration. In some instances (e.g., *A. exilispinous*), these color differences are apparent only during the courtship, while in others (e.g., *A. crockeri*, *A. aspera*) the sexes are permanently dichromatic in a number of features. The genus *Ekemblemaria* generally exhibits several uniquely evolved aspects of permanent coloration (see Hastings 1992a). Species in the *Chaenopsis* clade vary considerably in degree and pattern of sexual dichromatism within both *Chaenopsis* and *Emblemaria*.

All species exhibiting a high level of morphological dimorphism also exhibit a high degree of color dimorphism. Consequently, a pattern similar to that of morphological characters is seen in the combined data set in that the greatest degree of a dimorphism is seen in *Coralliozetus*, *Cirriemblemaria*, and most members of *Emblemaria* (Fig. 7).

## Discussion

The degree of sexual dimorphism documented in this survey represents minimum sexual differences for species of chaenopsids. Many species remain poorly known (Supplementary Table S2), only qualitative morphometric characters were included, and the breeding coloration of many species is unknown. In addition sex differences in bright colors were not included as these are known for only a few species and not retained in preserved museum specimens. Even with these limitations, chaenopsids exhibit an extraordinary range in degree of sexual dimorphism and an extraordinary level of dimorphism in several species. The group includes essentially monomorphic species as well as highly dimorphic species in which the sexes differ in over 30 phenotypic characters.

Members of the sister lineage to the remainder of the chaenopsids, *Neoclinus* and *Mccoskerichthys*, are relatively monomorphic with the sexes differing primarily in jaw length, albeit dramatically so in *N. blanchardi* (Hongjamrassilp et al. 2018). However the breeding coloration of these fishes has not been reported and the extent of dichromatism remains unknown. Although *Acanthemblemaria*, the most speciose genus in the family with 21 species, has undergone considerable morphological evolution in the pattern of spination on bones of the head (Smith-Vaniz and Palacio 1974; Hastings 1990), this has generally not involved divergence of the sexes. The common chaenopsid pattern of dimorphism in jaw length is reversed in this lineage, apparently through evolution of longer jaws in females, matching the jaw length of males (Fig. 8). Similar to many other fishes (Kodric-Brown 1998), most species of *Acanthemblemaria* exhibit distinct color differences related to reproduction when courting males increase in conspicuous coloration (e.g., Hastings 1988b). Also among the least dimorphic tube blennies are two species that are hypothesized mimics of other fishes. *Hemiemblemaria simulus*, the Wrasse Blenny, reportedly mimics the Bluehead Wrasse (Longley and Hildebrand 1940; but see Robertson 2003), while *L.zingaro*, the Arrow Blenny, resembles and swims with hovering gobies in the genus *Coryphopterus* (Greenfield 1972; Colin and Gomon 1973). The need to match the appearance of model species may constrain the divergence of males and females of these species. The Wrasse Blenny exhibits some variation in color pattern with growth (Longley and Hildebrand 1940; Stephens 1963; Böhlke and Chaplin 1968), but this variation has not been demonstrated to be associated with the sexes. Although no dimorphic characters have been identified for *H. simulus*, it is likely that males assume a unique coloration during courtship, consistent with that of other “monomorphic” chaenopsids such as *A. exilispinous* in which males temporarily darken during courtship, and *L. zingaro* in which males develop melanophores around the vent. The latter species is pedomorphic in several characters including jaw length (Fig. 8) which is shortened in both sexes compared with related chaenopsids (Hastings 1992b).

A moderate degree of dimorphism is seen in the speciose genera *Chaenopsis* and *Emblemariopsis*. These genera include both dimorphic and relatively monomorphic species implying considerable evolution within these apparently monophyletic lineages. However, females of several species of both genera are poorly known (Supplementary Table S2) and these groups may include more highly dimorphic species.

Three genera, *Emblemaria*, *Cirriemblemaria*, and *Coralliozetus*, exhibit the greatest degree of sexual dimorphism among chaenopsids (Table 3). Available evidence indicates that this level of sexual dimorphism evolved independently in each. First, different suites of dimorphic characters are observed in each genus. For example, several dimorphic features of *Coralliozetus* are unique to this genus including morphological features resulting from pedomorphosis in females (Hastings 2002). The reduction in sensory pore number on the dorsal surface of the head of males of this genus is caused by the occlusion of pore openings with growth as a consequence of the increased thickness of muscle insertion and increased fleshiness on the dorsal surface of the head (Hastings 1991a). Similarly, some species of *Emblemaria* are unique within chaenopsids in that the pelvic fin of males has a more elaborate and heavily pigmented inter-ray membrane than that of conspecific females and both sexes of other chaenopsids (see Smith et al. 1998, Fig. 4). Second, these genera are nested within different clades that include relatively monomorphic species (Fig. 7), thus independent evolution of extreme dimorphism is supported by the pattern of character mapping. Several of the dimorphic features of *Coralliozetus* are synapomorphies of this clade that apparently evolved in concert (Emerson and Hastings 1998; Hastings 2002). Similarly, dimorphic features of the monotypic *Cirriemblemaria* are not present in the related genera *Protemblemaria* and *Emblemariopsis*. The degree of dimorphism within *Emblemaria* is more variable, with most species being highly dimorphic, but others (e.g., *E. nivipes* and members of the “caldwelli species group”) being relatively monomorphic. However, several species of *Emblemaria* are poorly known (Supplementary Table S2) and the phylogenetic relationships of its included species have not been hypothesized based on shared derived features. Stephens (1963) considered *E. nivipes* to be the most “primitive” member of the genus, based in part on its relative lack of conspicuous dimorphism typical of many other species in the genus. However, this assertion has yet to be tested using cladistic methods. If his hypothesis is confirmed, it implies a more gradual evolution of the high degree of dimorphism in *Emblemaria* compared with the rapid evolution seen in *Cirriemblemaria* and *Coralliozetus* (Hastings 2002).

Why have some lineages of chaenopsids evolved such a striking degree of sexual dimorphism while others appear to have retained or reverted to monomorphism in morphological features? The answer lies, in part, with the pattern of microhabitat use by males and females as has been reported for sexual dimorphism in other groups (Shine 1989). Use of vacant tests of invertebrates is a hypothesized behavioral synapomorphy of the Chaenopsidae (Stephens 1963; Hastings and Springer 1994), but the pattern of shelter use varies within the family, and is an important key to understanding the selective pressures on chaenopsids. First, shelters serve as refuges from predators, and their availability may limit local populations densities (Buchheim and Hixon 1992; Hastings and Galland 2010). Second, shelters serve as egg deposition sites where eggs are fertilized and guarded by resident males. Successful defense of a high quality shelter, sometimes in limited supply (Hastings 1988a, 1992c), is necessary for male reproductive success (Hastings 1986), putting a premium on shelter defense. Shelter defense by males is facilitated by their generally robust features including heavily ossified bones of the head, long jaws, robust musculature, and large body size (Hastings 1991a).

Females of many chaenopsids also use shelters and may spend virtually all of their time inside of shelters (Hastings 2002). Evolution of sexual differences in morphology in these species may be constrained by the need for females to compete for shelters. In these, females are similar to males in having large, robust features. On the contrary, females of species of the highly dimorphic genus *Coralliozetus* (Hastings 2002) and the highly dimorphic *Emblemaria hypacanthus* (Hastings 1991b) spend significantly more time outside of shelters compared with conspecific males. In these species, females more closely resemble juveniles (i.e., are pedomorphic), differing from sexually mature males in having short jaws, relatively thin bones on the head, less well-developed musculature, and cryptic coloration. The more pointed pectoral fins of these females may also be related to their increased swimming behavior while residing in the open (Hastings 2002). One benefit for females abandoning shelter use is increased feeding rates compared with conspecifics residing in shelters; in some instances the number of bites taken by non-resident females is an order of magnitude greater than that of males residing in shelters (Hastings 2002). Higher feeding rates are also seen in juveniles and males residing outside of shelters as a consequence of their ability to forage over a wider area compared with individuals restricted to shelters (Hastings 2002). It is likely that a similar divergence in time spent inside versus outside of a shelter is related to the increased dimorphism in *C.lucasana* and some species of *Emblemariopsis* (see Tyler and Tyler 1999).

Sexual differences in habitat use do not appear to be the only factor involved in the evolution of sexual dimorphism in chaenopsids. While competition for shelters dictates robust morphology including long jaws, many features of males of highly dimorphic species are more characteristic of epigamic selection (Anderson 1984; Prum 2017). This is especially true of the elaborate morphology of males and the conspicuous coloration they assume when courting females. In several species, dimorphic features of males such as the elevated dorsal fin and elongate supraorbital cirri are accentuated by bright coloration (features not included in this study). For example, while female chaenopsids typically have drab supraorbital cirri, courting males of species of *Emblemaria* have cirri ranging from black to red to blue, sometimes with bands of color, while courting males of species of *Coralliozetus* have cirri ranging from black (*C. micropes* and *C. cardonae*; Fig. 3C) to white (*C. rosenblatti* and *C. boehlkei*; Fig. 3E) to blue (*C. springeri*) to yellow (*C. angelicus*; personal observations). Also, males are more likely than females to exhibit bright colors on the anterior dorsal fin, a region prominently displayed during courtship (Fig. 2E,F).

The features of chaenopsids that exhibit the greatest differences between the sexes are generally located on the head and anterior dorsal fin (Table 1). These areas are particularly important in aggressive and courtship displays in chaenospids and other blennies (Thresher 1984; Neat and Lengkeek 2009). In aggressive interactions, resident tube blennies extend partially outside of their shelter, flare the branchiostegal membranes, erect the dorsal fin, and often gape the mouth. Similarly, these same areas are especially evident in males during courtship which typically involves a male lunging outward from the shelter, erecting the dorsal fin and flaring the branchial region (personal observations). All of these are areas of the head and body that show considerable variation in morphology and in coloration, both across species and across the sexes of many chaenopsids.

Simply mapping the presence/absence of sexual dimorphism (Figs. 5–7) masks considerable evolution. This is illustrated by looking at the evolution of relative jaw length in males and females. Mapping the presence/absence of dimorphism in jaw length indicates that the sexes diverged at least four times assuming a minimum number of steps within polytomies (Fig. 8A). However, mapping relative jaw length in males and females separately indicates a substantially higher rate of evolution in both sexes (Fig. 8B,C). This included co-evolution of jaw length in both sexes to maintain monomorphism (e.g., elongation in *Chaenopsis*, reduction in *Lucayablennius*), evolution in a single sex to achieve monomorphism (e.g., elongation in females of *Protemblemaria*), and evolution in one sex to achieve or magnify dimorphism (e.g., reduction in females of *Emblemaria*, elongation in males of two species of *Neoclinus*).

Sexual dimorphism in jaw length is seen in a variety of non-hermaphroditic teleosts including salmonids (e.g., Beacham and Murray 1986), rockfishes (Lenarz and Echeveria 1991), gymnotiforms (Hilton and Fernandez 2006), gobies (e.g., Crabtree 1985; Pezold 2004), and other blenniiforms (e.g., Kotrschal 1988; Brooks 1991). Longer jaws in males is also common in oral brooding fishes such as jawfishes (Smith-Vaniz 1972), cardinal fishes (Barnett and Bellwood 2005), and cichlids (Oliveira and Almada 1995) that likely facilitate accommodation of increased numbers or improved development of eggs (Hess 1993; Barnett and Bellwood 2005). Some authors have suggested that jaw dimorphism in fishes may be related to partitioning of food resources by the sexes, but this rarely has been demonstrated (but see McGee and Wainwright 2003), and has not been examined in chaenopsids or other blenniiforms (Kotrschal and Thomson 1986). In dimorphic chaenopsids, the jaws of males are invariably longer than those of females, a situation common to most fishes exhibiting jaw dimorphism. Available evidence supports the hypothesis that increased jaw size of males in at least one blenny serves to amplify apparent body size during aggressive gaping displays (Hongjamrassilp et al. 2018).

Chaenopsids join a growing list of groups for which a complex pattern of evolution of dimorphism is emerging involving repeated trait reversals (Omland 1997; Wiens 2001) and divergence in females as well as in males (e.g., Gluckman 2014; Price and Eaton 2014; Price 2015). It is clear that simply coding characters as dimorphic or not dimorphic potentially obscures a more complex evolutionary history within lineages. A more complete understanding of the evolution of sexual differences in groups such as the Chaenopsidae will require study of the evolution of single characters independently in both males and females on fully resolved phylogenetic hypotheses.

## Summary

The strikingly different patterns of sexual dimorphism exhibited by chaenopsids have come about via complex patterns of evolution including changes solely in males, solely in females, and simultaneous changes in both sexes. This represents an ideal group to study selective factors leading to the divergence of the sexes. This is especially true because the mating system of chaenopsids, a resource defense polygyny system with male care of eggs is common to both relatively monomorphic and highly dimorphic species (Hastings 1986; Hastings and Petersen 2010). The underlying causes for the evolution of dimorphism identified to date include habitat segregation (Hastings 2002), but also clearly involve both forms of sexual selection.

## Supplementary Material

obz003_Supplementary_Material
