# Supplementary material for: Evolution of Sexual Dimorphism in Tube Blennies (Teleostei: Chaenopsidae)
Source: Integr Org Biol. 2019 Mar 6;1(1):obz003. doi: 10.1093/iob/obz003 (PMC7671137; doi:10.1093/iob/obz003)
Supplement: obz003_Supplementary_Material [file obz003_supplementary_material.zip › obz003 Table S1 Corrected.docx]

Table S1. Specimens examined. Collection abbreviations follow Sabaj (2016); PAH = P.A. Hastings uncataloged collections.

_____________________________________________________________________________________________________________________

Genus Species Specimens examined

_____________________________________________________________________________________________________________________

| *Acanthemblemaria* | *aspera* | ANSP 121873; FMNH 93967; FMNH 93968; FMNH 93974; SIO 01-09; SIO 03-150 |
| --- | --- | --- |
| *Acanthemblemaria* | *atrata* | LACM 32256; LACM 32271; LACM 32283; UAZ 89-1; USNM 346390; USNM 346391 |
| *Acanthemblemaria* | *balanorum* | SIO 62-55; UAZ 71-61; UAZ 73-117; UAZ 74-48; UAZ 77-20; USNM 317628 |
| *Acanthemblemaria* | *betinensis* | ANSP 122851; SIO 03-141; SIO 71-262 |
| *Acanthemblemaria* | *castroi* | LACM 56862; SIO 64-1003; USNM 317701 |
| *Acanthemblemaria* | *chaplini* | ANSP 100882; ANSP 144938; SIO 03-141 |
| *Acanthemblemaria* | *crockeri* | PAH 8419; UAZ 71-36; UAZ 74-57; USNM 317658 |
| *Acanthemblemaria* | *exilispinis* | ANSP 102508; LACM 32500; LACM 32548; SIO 70-356; UAZ 68-74; USNM 317708 |
| *Acanthemblemaria* | *greenfieldi* | FMNH 90493; SIO 03-145; SIO 03-151 |
| *Acanthemblemaria* | *hancocki* | GCRL 3552; LACM 315794; LACM 32566; UAZ 68-73; UAZ 85-17 |
| *Acanthemblemaria* | *harpeza* | SIO 02-79 |
| *Acanthemblemaria* | *hastingsi* | SIO 00-14; SIO 65-341; UAZ 73-117; UAZ 77-20; UAZ 77-42 |
| *Acanthemblemaria* | *macrospilus* | UAZ 70-22; UAZ 71-63; UAZ 73-66; UAZ 77-41; USNM 317623; USNM 317625; USNM 317626 |
| *Acanthemblemaria* | *mangognatha* | UAZ 90-1; USNM 346392; USNM 346393 |
| *Acanthemblemaria* | *maria* | AMNH 24196; ANSP 144939; SIO 03-141; SIO 03-147 |
| *Acanthemblemaria* | *medusa* | ANSP 113963; ANSP 122844 |
| *Acanthemblemaria* | *paula* | FMNH 90876; SIO 03-145; SIO 03-146; SIO 89-13 |
| *Acanthemblemaria* | *rivasi* | ANSP 147650; SIO 03-141; SIO 71-272; SIO 71-286 |
| *Acanthemblemaria* | *spinosa* | ANSP 121872; ANSP 143021; SIO 03-150; SIO 03-157 |
| *Acanthemblemaria* | *stephensi* | SIO 72-96; SIO 72-97 |
| *Chaenopsis* | *alepidota* | SIO 62-212; SIO 65-351 |
| *Chaenopsis* | *coheni* | SIO 61-247; SIO 61-249; SIO 61-277; SIO 65-295 |
| *Chaenopsis* | *deltarrhis* | USNM 317713; USNM 317714 |
| *Chaenopsis* | *limbaughi* | ANSP 120970; SIO 03-149; SIO 03-150 |
| *Chaenopsis* | *megalops* | ANSP 138519 |
| *Chaenopsis* | *ocellata* | ANSP 134977; ANSP 141707 |
| *Chaenopsis* | *resh* | ANSP 102732 |
| *Chaenopsis* | *schmitti* | CAS 46585; CAS 46587; CAS 48732; CAS 50095; CAS 214774; CAS 214881 |
| *Chaenopsis* | n. sp. | SIO 67-40; USNM 31675; USNM 31685; USNM 317712 |
| *Cirriemblemaria* | *lucasana* | SIO 65-317; SIO 65-343; SIO 65-347; SIO 65-354; UAZ 70-23; UAZ 72-87; UAZ 73-94 |
| *Coralliozetus* | *angelicus* | UAZ 77-20; UAZ 77-42; SIO 01-182; SIO 59-210; SIO 61-225; SIO 61-232; SIO 65-341 |
| *Coralliozetus* | *boehlkei* | UAZ 69-48; UAZ 77-41; UAZ 77-44 |
| *Coralliozetus* | *cardonae* | ANSP 113946; ANSP 144996 |
| *Coralliozetus* | *micropes* | CAS 58524; SIO 65-331; UAZ 71-36; UAZ 74-36 |
| *Coralliozetus* | *rosenblatti* | LACM 32086; SIO 61-225; SIO 61-256; UAZ 85-8 |
| *Coralliozetus* | *springeri* | SIO 01-164; SIO 67-34 |
| *Ekemblemaria* | *myersi* | PAH 8222; SIO 59-225; UAZ 69-48; UAZ 72-105; UAZ 82-21 |
| *Ekemblemaria* | *nigra* | SIO 67-45; UMML 22455 |
| *Emblemaria* | *atlantica* | ANSP 133225; ANSP 133227; UF 30561; UWF 3666 |
| *Emblemaria* | *caldwelli* | FMNH 87922; UF 23391; UF 24410; USNM 267828 |
| *Emblemaria* | *caycedoi* | UF 25826; UF 32669; UMML 30147 |
| *Emblemaria* | *diphyodontis* | SIO 04-164; SIO 04-168 |
| *Emblemaria* | *hudsoni* | CAS 58500; USNM 122023; USNM 128220 |
| *Emblemaria* | *hyltoni* | FMNH 96793; FMNH 96856; FMNH 96865; FMNH 96876 |
| *Emblemaria* | *hypacanthus* | PAH 8315; SIO 61-279; SIO 62-212; SIO 65-337; SIO 74-124 |
| *Emblemaria* | *nivipes* | CAS 154144; SIO 01-46; SIO 01-165; USNM 094020; USNM 101935 |
| *Emblemaria* | *pandionis* | ANSP 115119; ANSP 143027; PAH 7766; SIO 01-9; SIO 03-149; SIO 03-151 |
| *Emblemaria* | *piratica* | SIO 01-182; SIO 62-49; USNM 317678; USNM 317679; USNM 317715; USNM 317716 |
| *Emblemaria* | *piratula* | ANSP 136248; ANSP 143826; UWF 1388 |
| *Emblemaria* | *walkeri* | SIO 62-216; SIO 62-218; SIO 74-124 |
| *Emblemariopsis* | *bahamensis* | ANSP 114004; ANSP 114007; ANSP 114009 |
| *Emblemariopsis* | *diaphana* | UF 10850; UF 11882; UMML 11670; UMML 11671 |
| *Emblemariopsis* | *leptocirris* | ANSP 113998; ANSP 114014; LACM 8939-4; UF 24750 |
| *Emblemariopsis* | *occidentalis* | ANSP 74772; ANSP 113982; ANSP 114004; ANSP 168607; UMML 30374 |
| *Emblemariopsis* | *pricei* | FMNH 90513; FMNH 94299; FMNH 96803; SIO 03-148; SIO 03-151 |
| *Emblemariopsis* | *randalli* | SIO 04-177; SIO 06-276 |
| *Emblemariopsis* | *signifera* | FMNH 87929; FMNH 87930; FMNH 94001; SIO 01-171; USNM 199622 |
| *Hemiemblemaria* | *simulus* | FMNH 86171; FMNH 90522 |
| *Lucayablennius* | *zingaro* | FMNH 96759 |
| *Mccoskerichthys* | *sandae* | SIO 70-359; SIO 75-404 |
| *Neoclinus* | *blanchardi* | SIO 47-183; SIO 71-163; SIO 85-14 |
| *Neoclinus* | *stephensae* | LACM 38626; SIO 56-16; SIO 59-307; SIO 70-130 |
| *Neoclinus* | *uninotatus* | SIO 10-82; SIO 68-585; SIO 69-310 |
| *Protemblemaria* | *bicirris* | SIO 00-14; SIO 00-15; SIO 62-105; SIO 71-260; USNM 317683; USNM 317705 |
| *Protemblemaria* | *perla* | SIO 00-64; SIO 14-35; SIO 78-13; USNM 353939; USNM 353940 |
| *Protemblemaria* | *punctata* | ANSP 103565; UMML 19245 |
