# Supplementary material for: Evolution of Sexual Dimorphism in Tube Blennies (Teleostei: Chaenopsidae)
Source: Integr Org Biol. 2019 Mar 6;1(1):obz003. doi: 10.1093/iob/obz003 (PMC7671137; doi:10.1093/iob/obz003)
Supplement: obz003_Supplementary_Material [file obz003_supplementary_material.zip › Table S2 Omitted species final.docx]

Table S2. Species of chaenopsids not included in this survey.

____________________________________________________________________________

Species Comments

____________________________________________________________________________

*Acanthemblemaria johnsoni* Almay & Baldwin Females unavailable for this study

*Chaenopsis megalops* Smith-Vaniz Known from only two males

*Chaenopsis stephensi* Robins & Randall Known from only one female

*Ekemblemaria lira* Hastings Known from two males & one female

*Emblemaria australis* Ramos, Rocha & Rocha Females unavailable for this study

*Emblemaria biocellata* Stephens Known from two males & one female

*Emblemaria culmensis* Stephens Known from only one male

*Emblemaria vitta* Williams Known from only males

*Emblemariopsis arawak* Victor Females unavailable for this study

*Emblemariopsis bottomei* Stephens Known from only one male

*Emblemariopsis carib* Victor Females unavailable for this study

*Emblemariopsis ruetzleri* Tyler & Tyler Females unavailable for this study

*Emblemariopsis tayrona* Acero Females unavailable for this study

*Emblemariopsis ramirezi* Cervigon Known from only males

*Emblemariopsis dianae* Tyler & Hastings Known from only males

*Neoclinus* western Pacific clade (8 species) Specimens unavailable for this study

*Tanyemblemaria alleni* Hastings Known from only one male

____________________________________________________________________________
