# Supplementary material for: Evolution of Sexual Dimorphism in Tube Blennies (Teleostei: Chaenopsidae)
Source: Integr Org Biol. 2019 Mar 6;1(1):obz003. doi: 10.1093/iob/obz003 (PMC7671137; doi:10.1093/iob/obz003)
Supplement: obz003_Supplementary_Material [file obz003_supplementary_material.zip › Table S3 Scores for all species.pdf]

	M-Ia. Infraorbital size	M-Ib. Infraorbital number	M-Ic. Infraorbital texture	M-Id. Nasal bones	M-Ie. Nasal bone texture	M-IIa. Head shape	M-IIb. Jaw length
Acanthemblemaria							
aspera M/F	thick/thick	two/two	smooth/smooth	fused/fused	smooth/smooth	rounded/rounded	> PO/> PO
atrata M/F	thick/thick	two/two	spines/spines	fused/fused	spines/spines	rounded/rounded	> PO/> PO
balanorum M/F	thick/thick	two/two	spines/spines	fused/fused	spines/spines	rounded/rounded	> PO/> PO
betinensis M/F	thick/thick	two/two	spines/spines	fused/fused	spines/spines	rounded/rounded	> PO/> PO
castroi M/F	thick/thick	two/two	ridges/ridges	fused/fused	spines/spines	rounded/rounded	> PO/> PO
chaplini M/F	thick/thick	two/two	smooth/smooth	fused/fused	smooth/smooth	rounded/rounded	> PO/> PO
crockeri M/F	thick/thick	two/two	spines/spines	fused/fused	spines/spines	rounded/rounded	> PO/> PO
exilispinis M/F	thick/thick	two/two	spines/spines	fused/fused	spines/spines	rounded/rounded	> PO/> PO
greenfieldi M/F	thick/thick	two/two	smooth/smooth	fused/fused	smooth/smooth	rounded/rounded	> PO/> PO
hancocki M/F	thick/thick	two/two	spines/spines	fused/fused	spines/spines	rounded/rounded	> PO/> PO
harpezi M/F	thick/thick	two/two	spines/spines	fused/fused	spines/spines	rounded/rounded	> PO/> PO
hastingsi M/F	thick/thick	two/two	spines/spines	fused/fused	spines/spines	rounded/rounded	> PO/> PO
macrospilus M/F	thick/thick	two/two	spines/spines	fused/fused	spines/spines	rounded/rounded	> PO/> PO
mangognatha M/F	thick/thick	two/two	spines/spines	fused/fused	spines/spines	rounded/rounded	> PO/> PO
maria M/F	thick/thick	two/two	spines/spines	fused/fused	spines/spines	rounded/rounded	> PO/> PO
medusa M/F	thick/thick	two/two	spines/spines	fused/fused	smooth/smooth	rounded/rounded	> PO/> PO
paula M/F	thick/thick	two/two	spines/spines	fused/fused	spines/spines	rounded/rounded	> PO/> PO
rivasi M/F	thick/thick	two/two	ridges/ridges	fused/fused	spines/spines	rounded/rounded	> PO/> PO
spinosa M/F	thick/thick	two/two	spines/spines	fused/fused	spines/spines	rounded/rounded	> PO/> PO
stephensi M/F	thick/thick	two/two	spines/spines	fused/fused	spines/spines	rounded/rounded	> PO/> PO
Chaenopsis							
alepidota M/F	slender/slender	two/two	pits/pits	separate/separate	pits/pits	elongate/elongate	>> PO/>> PO
coheni M/F	slender/slender	two/two	pits/pits	separate/separate	pits/pits	elongate/elongate	>> PO/>> PO
deltarrhis M/F	slender/slender	two/two	pits/pits	separate/separate	pits/pits	elongate/elongate	>> PO/>> PO
limbaughi M/F	slender/slender	two/two	pits/pits	separate/separate	pits/pits	elongate/elongate	>> PO/>> PO
ocellata M/F	slender/slender	two/two	pits/pits	separate/separate	pits/pits	elongate/elongate	>> PO/>> PO
resh M/F	slender/slender	two/two	pits/pits	separate/separate	pits/pits	elongate/elongate	>> PO/>> PO
roseola M/F	slender/slender	two/two	pits/pits	separate/separate	pits/pits	elongate/elongate	> PO/> PO
schmitti M/F	slender/slender	two/two	pits/pits	separate/separate	pits/pits	elongate/elongate	> PO/> PO
new species. M/F	slender/slender	two/two	pits/pits	separate/separate	pits/pits	elongate/elongate	> PO/> PO
Cirriemblemaria							
lucasana M/F	* thick/slender	two/two	* pits/smooth	separate/separate	* pits/smooth	rounded/rounded	* > PO/< PO
Coralliozetus							
angelicus M/F	* thick/slender	* two/three	* pits/smooth	* fused/separate	* pits/smooth	* rounded/pointed	* > PO/< PO
boehlkei M/F	* thick/slender	* two/three	* pits/smooth	* fused/separate	* pits/smooth	* rounded/pointed	* > PO/< PO
cardonae M/F	* thick/slender	* two/three	* pits/smooth	* fused/separate	* pits/smooth	* rounded/pointed	* > PO/< PO
micropes M/F	* thick/slender	* two/three	* pits/smooth	* fused/separate	* pits/smooth	* rounded/pointed	* > PO/< PO
rosenblatti M/F	* thick/slender	* two/three	* pits/smooth	* fused/separate	* pits/smooth	* rounded/pointed	* > PO/< PO
springeri M/F	* thick/slender	* two/three	* pits/smooth	* fused/separate	* pits/smooth	* rounded/pointed	* > PO/< PO
Ekemblemaria							
myersi M/F	thick/thick	two/two	pits/pits	fused/fused	pits/pits	rounded/rounded	> PO/> PO
nigra M/F	thick/thick	two/two	pits/pits	fused/fused	pits/pits	rounded/rounded	> PO/> PO

Emblemaria							
atlantica M/F	* thick/slender	two/two	* pits/smooth	separate/separate	* high ridge/low ridge	* rounded/pointed	* > PO/PO
caldwelli M/F	slender/slender	two/two	smooth/smooth	separate/separate	ridge/ridge	pointed/pointed	PO/PO
caycedoi M/F	slender/slender	two/two	smooth/smooth	separate/separate	ridge/ridge	rounded/rounded	> PO/> PO
diphyodontis M/F	* thick/slender	two/two	* pits/smooth	separate/separate	* knobs/smooth	rounded/rounded	* > PO/PO
hudsoni M/F	* thick/slender	two/two	* pits/smooth	separate/separate	* high ridge/low ridge	* rounded/pointed	* > PO/PO
hyltoni M/F	slender/slender	two/two	smooth/smooth	separate/separate	ridge/ridge	pointed/pointed	PO/PO
hypacanthus M/F	* thick/slender	two/two	* pits/smooth	separate/separate	* high ridge/low ridge	* rounded/pointed	* > PO/PO
nivipis M/F	thick/thick	two/two	pits/pits	separate/separate	ridge/ridge	rounded/rounded	> PO/> PO
pandionis M/F	thick/thick	two/two	smooth/smooth	separate/separate	ridge/ridge	rounded/rounded	> PO/> PO
piratica M/F	* thick/slender	two/two	* pits/smooth	separate/separate	* knobs/smooth	rounded/rounded	* > PO/PO
piratula M/F	* thick/slender	two/two	* pits/smooth	separate/separate	ridge/ridge	pointed/pointed	PO/PO
walkeri M/F	* thick/slender	two/two	* pits/smooth	separate/separate	* pits/smooth	* rounded/pointed	* > PO/PO
Emblemariopsis							
bahamensis M/F	* thick/slender	two/two	* pits/smooth	separate/separate	* pits/smooth	* rounded/pointed	* PO/< PO
diaphana M/F	* thick/slender	two/two	smooth/smooth	separate/separate	smooth/smooth	rounded/rounded	* PO/< PO
leptocirris M/F	* thick/slender	two/two	smooth/?	separate/separate	smooth/smooth	* rounded/pointed	* > PO/ PO
occidentalis M/F	* thick/slender	two/two	* pits/smooth	separate/separate	smooth/smooth	* rounded/pointed	* PO/< PO
pricei M/F	* thick/slender	two/two	?/?	separate/separate	smooth/smooth	rounded/rounded	PO/PO
randalli M/F	* thick/slender	two/two	?/?	separate/separate	smooth/smooth	rounded/rounded	* > PO/< PO
signifera M/F	* thick/slender	two/two	* pits/smooth	separate/separate	smooth/smooth	pointed/pointed	* PO/< PO
Hemiemblemaria							
simulus M/F	slender/slender	two/two	pits/pits	separate/separate	smooth/smooth	pointed/pointed	> PO/> PO
Lucayablennius							
zingaro M/F	slender/slender	two/two	smooth/smooth	separate/separate	smooth/smooth	pointed/pointed	< PO/< PO
Mccoskerichthys							
sandae M/F	thick/thick	four/four	pits/pits	fused/fused	smooth/smooth	rounded/rounded	* PO/< PO
Neoclinus							
blanchardi M/F	thick/thick	four/four	smooth/smooth	separate/separate	smooth/smooth	rounded/rounded	* >> POP/ > POP
stephensae M/F	thick/thick	four/four	smooth/smooth	separate/separate	smooth/smooth	rounded/rounded	> PO/> PO
uninotatus M/F	thick/thick	four/four	smooth/smooth	separate/separate	smooth/smooth	rounded/rounded	* POP/< POP
Protemblemaria							
bicirris M/F	thick/thick	two/two	pits/pits	fused/fused	few pits/few pits	rounded/rounded	> PO/> PO
perla M/F	thick/thick	two/two	pits/pits	fused/fused	few pits/few pits	rounded/rounded	> PO/> PO
punctata MF	thick/thick	two/two	pits/pits	fused/fused	few pits/few pits	rounded/rounded	> PO/> PO

	M-IIc. Supraorbital cirris length	M-IId. Nasal cirris length	M-IIIa. Lip shape	M-IIIb. Dewlap	M-IIIc. Snout flaps	M-IIId. Nape papillae	IIIe.M- Nape folds	M-IIIf. Nape muscles
Acanthemblemaria								
aspera M/F	1 orbit/1 orbit	moderate/moderate	flat/flat	absent/absent	absent/absent	absent/absent	absent/absent	present/present
atrata M/F	1/2-1 orbit/1/2-1 orbit	long/long	flat/flat	absent/absent	absent/absent	absent/absent	absent/absent	present/present
balanorum M/F	1 orbit/1 orbit	moderate/moderate	flat/flat	absent/absent	absent/absent	absent/absent	absent/absent	present/present
betinensis M/F	1 orbit/1 orbit	moderate/moderate	flat/flat	absent/absent	absent/absent	absent/absent	absent/absent	present/present
castroi M/F	1 orbit/1 orbit	moderate/moderate	flat/flat	absent/absent	absent/absent	absent/absent	absent/absent	present/present
chaplini M/F	1 orbit/1 orbit	moderate/moderate	flat/flat	absent/absent	absent/absent	absent/absent	absent/absent	present/present
crockeri M/F	1 orbit/1 orbit	moderate/moderate	flat/flat	absent/absent	absent/absent	absent/absent	absent/absent	present/present
exilispinis M/F	1 orbit/1 orbit	moderate/moderate	flat/flat	absent/absent	absent/absent	absent/absent	absent/absent	present/present
greenfieldi M/F	1 orbit/1 orbit	moderate/moderate	flat/flat	absent/absent	absent/absent	absent/absent	absent/absent	present/present
hancocki M/F	1/2-1 orbit/1/2-1 orbit	moderate/moderate	flat/flat	absent/absent	absent/absent	absent/absent	absent/absent	present/present
harpezi M/F	1/2-1 orbit/1/2-1 orbit	long/long	flat/flat	absent/absent	absent/absent	absent/absent	absent/absent	present/present
hastingsi M/F	1 orbit/1 orbit	moderate/moderate	flat/flat	absent/absent	absent/absent	absent/absent	absent/absent	present/present
macrospilus M/F	1 orbit/1 orbit	moderate/moderate	flat/flat	absent/absent	absent/absent	absent/absent	absent/absent	present/present
mangognatha M/F	1 orbit/1 orbit	moderate/moderate	flat/flat	absent/absent	absent/absent	absent/absent	absent/absent	present/present
maria M/F	1 orbit/1 orbit	moderate/moderate	flat/flat	absent/absent	absent/absent	absent/absent	absent/absent	present/present
medusa M/F	1 orbit/1 orbit	moderate/moderate	flat/flat	absent/absent	absent/absent	absent/absent	absent/absent	present/present
paula M/F	* 1/2-1 orbit/< 1/2-1 orbit	* moderate/short	flat/flat	absent/absent	absent/absent	absent/absent	absent/absent	present/present
rivasi M/F	1/2-1 orbit/1/2-1 orbit	moderate/moderate	flat/flat	absent/absent	absent/absent	absent/absent	absent/absent	present/present
spinosa M/F	1 orbit/1 orbit	moderate/moderate	flat/flat	absent/absent	absent/absent	absent/absent	absent/absent	present/present
stephensi M/F	1/2-1 orbit/1/2-1 orbit	moderate/moderate	flat/flat	absent/absent	absent/absent	absent/absent	absent/absent	present/present
Chaenopsis								
alepidota M/F	absent/absent	absent/absent	flat/flat	absent/absent	absent/absent	absent/absent	absent/absent	present/present
coheni M/F	absent/absent	absent/absent	flat/flat	absent/absent	absent/absent	absent/absent	absent/absent	present/present
deltarrhis M/F	absent/absent	absent/absent	flat/flat	absent/absent	absent/absent	absent/absent	absent/absent	present/present
limbaughi M/F	absent/absent	absent/absent	flat/flat	* large/small	absent/absent	absent/absent	absent/absent	present/present
ocellata M/F	absent/absent	absent/absent	flat/flat	large/large	absent/absent	absent/absent	absent/absent	present/present
resh M/F	absent/absent	absent/absent	flat/flat	* large/small	absent/absent	absent/absent	absent/absent	present/present
roseola M/F	absent/absent	absent/absent	flat/flat	absent/absent	absent/absent	absent/absent	absent/absent	present/present
schmitti M/F	absent/absent	absent/absent	flat/flat	absent/absent	absent/absent	absent/absent	absent/absent	present/present
new species. M/F	absent/absent	absent/absent	flat/flat	absent/absent	absent/absent	absent/absent	absent/absent	present/present
Cirriemblemaria								
lucasana M/F	* >> orbit/1 orbit	* long/moderate	flat/flat	absent/absent	* present/absent	* many/few	absent/absent	* present/absent
Coralliozetus								
angelicus M/F	* > orbit/<< orbit	* moderate/short	* flat/protruding	absent/absent	absent/absent	absent/absent	absent/absent	* present/absent
boehlkei M/F	* > orbit/<< orbit	* moderate/short	* flat/protruding	absent/absent	absent/absent	absent/absent	absent/absent	* present/absent
cardonae M/F	* > orbit/<< orbit	* moderate/short	* flat/protruding	absent/absent	absent/absent	absent/absent	absent/absent	* present/absent
micropes M/F	* > orbit/<< orbit	* moderate/short	* flat/protruding	absent/absent	absent/absent	absent/absent	absent/absent	* present/absent
rosenblatti M/F	<< orbit/<< orbit	* moderate/short	* flat/protruding	absent/absent	absent/absent	absent/absent	absent/absent	* present/absent
springeri M/F	* > orbit/<< orbit	* moderate/short	* flat/protruding	absent/absent	absent/absent	absent/absent	absent/absent	* present/absent
Ekemblemaria								
myersi M/F	* > orbit/< orbit	moderate/moderate	flat/flat	absent/absent	absent/absent	absent/absent	absent/absent	present/present
nigra M/F	* > orbit/< orbit	moderate/moderate	flat/flat	absent/absent	absent/absent	absent/absent	absent/absent	present/present

Emblemaria									
atlantica M/F	* >> orbit/< orbit	* very long/long	flat/flat	absent/absent	absent/absent	absent/absent	* present/absent	present/present	
caldwelli M/F	* > orbit/< orbit	long/long	flat/flat	absent/absent	absent/absent	absent/absent	absent/absent	present/present	
caycedoi M/F	* > orbit/< orbit	moderate/moderate	flat/flat	absent/absent	absent/absent	absent/absent	* present/absent	present/present	
diphyodontis M/F	* 1 orbit/< 1/2 orbit	* long/short	flat/flat	absent/absent	absent/absent	absent/absent	* present/absent	present/present	
hudsoni M/F	* >> orbit/< orbit	* moderate/short	flat/flat	absent/absent	absent/absent	absent/absent	* present/absent	present/present	
hyltoni M/F	* >> orbit/> orbit	* very long/long	flat/flat	absent/absent	absent/absent	absent/absent	absent/absent	present/present	
hypacanthus M/F	* 1 orbit/< 1/2 orbit	* very long/long	flat/flat	absent/absent	absent/absent	absent/absent	* present/absent	present/present	
nivipis M/F	1 orbit/1 orbit	long/long	flat/flat	absent/absent	absent/absent	absent/absent	absent/absent	present/present	
pandionis M/F	* 1 orbit/< orbit	moderate/moderate	flat/flat	absent/absent	absent/absent	absent/absent	absent/absent	present/present	
piratica M/F	* 1/2 orbit/< 1/2 orbit	* moderate/short	flat/flat	absent/absent	absent/absent	absent/absent	* present/absent	present/present	
piratula M/F	* >> orbit/<< 1/2 orbit	* moderate/tiny	flat/flat	absent/absent	absent/absent	absent/absent	absent/absent	present/present	
walkeri M/F	* 1 orbit/< orbit	short/short	flat/flat	absent/absent	absent/absent	absent/absent	* present/absent	present/present	
Emblemariopsis									
bahamensis M/F	absent/absent	moderate/moderate	flat/flat	absent/absent	absent/absent	absent/absent	absent/absent	* present/absent	
diaphana M/F	absent/absent	moderate/moderate	flat/flat	absent/absent	absent/absent	absent/absent	absent/absent	present/present	
leptocirris M/F	tiny/tiny	moderate/moderate	flat/flat	absent/absent	absent/absent	absent/absent	absent/absent	present/?	
occidentalis M/F	tiny/tiny	short/short	flat/flat	absent/absent	absent/absent	absent/absent	absent/absent	* present/absent	
pricei M/F	absent/absent	short/short	flat/flat	absent/absent	absent/absent	absent/absent	absent/absent	present/?	
randalli M/F	absent/absent	short/short	flat/flat	absent/absent	absent/absent	absent/absent	absent/absent	* present/absent	
signifera M/F	tiny/tiny	short/short	flat/flat	absent/absent	absent/absent	absent/absent	absent/absent	* present/absent	
Hemiemblemaria									
simulus M/F	absent/absent	short/short	flat/flat	absent/absent	absent/absent	absent/absent	absent/absent	present/present	
Lucayablennius									
zingaro M/F	absent/absent	absent/absent	flat/flat	absent/absent	absent/absent	absent/absent	absent/absent	absent/absent	
Mccoskerichthys									
sandae M/F	1 orbit/1 orbit	moderate/moderate	flat/flat	absent/absent	absent/absent	absent/absent	absent/absent	absent/absent	
Neoclinus									
blanchardi M/F	1 orbit/1 orbit	moderate/moderate	flat/flat	absent/absent	absent/absent	absent/absent	absent/absent	absent/absent	
stephensae M/F	* > orbit/1 orbit	long/long	flat/flat	absent/absent	absent/absent	absent/absent	absent/absent	absent/absent	
uninotatus M/F	* >> orbit/1 orbit	long/long	flat/flat	absent/absent	absent/absent	absent/absent	absent/absent	absent/absent	
Protemblemaria									
bicirris M/F	1 orbit/1 orbit	moderate/moderate	flat/flat	absent/absent	absent/absent	absent/absent	absent/absent	present/present	
perla M/F	1 orbit/1 orbit	moderate/moderate	flat/flat	absent/absent	absent/absent	absent/absent	present/present	present/present	
punctata MF	1 orbit/1 orbit	moderate/moderate	flat/flat	absent/absent	absent/absent	absent/absent	present/present	present/present	

	M-IIIg. Nape sensory pores	M-IVa. DF shape	M-IVb. DF height	M-IVc. DF flap	M-IVd. DF notch	M-IVe. Pectoral fin shape
Acanthemblemaria						
aspera M/F	same/same	even/even	low/low	thin/thin	absent/absent	rounded/rounded
atrata M/F	same/same	even/even	low/low	absent/absent	slight/slight	rounded/rounded
balanorum M/F	same/same	even/even	low/low	thin/thin	slight/slight	rounded/rounded
betinensis M/F	same/same	even/even	low/low	thin/thin	absent/absent	rounded/rounded
castroi M/F	same/same	even/even	low/low	absent/absent	slight/slight	rounded/rounded
chaplini M/F	same/same	even/even	low/low	thin/thin	absent/absent	rounded/rounded
crockeri M/F	same/same	even/even	low/low	thin/thin	slight/slight	rounded/rounded
exilispinis M/F	same/same	even/even	low/low	thin/thin	absent/absent	rounded/rounded
greenfieldi M/F	same/same	even/even	low/low	thin/thin	absent/absent	rounded/rounded
hancocki M/F	same/same	even/even	low/low	absent/absent	slight/slight	rounded/rounded
harpezi M/F	same/same	even/even	low/low	thin/thin	absent/absent	rounded/rounded
hastingsi M/F	same/same	even/even	low/low	absent/absent	slight/slight	rounded/rounded
macrospilus M/F	same/same	even/even	low/low	absent/absent	slight/slight	rounded/rounded
mangognatha M/F	same/same	even/even	low/low	absent/absent	slight/slight	rounded/rounded
maria M/F	same/same	even/even	low/low	thin/thin	absent/absent	rounded/rounded
medusa M/F	same/same	even/even	low/low	thin/thin	absent/absent	rounded/rounded
paula M/F	same/same	even/even	low/low	thin/thin	absent/absent	rounded/rounded
rivasi M/F	same/same	even/even	low/low	absent/absent	slight/slight	rounded/rounded
spinosa M/F	same/same	even/even	low/low	thin/thin	absent/absent	rounded/rounded
stephensi M/F	same/same	even/even	low/low	absent/absent	slight/slight	rounded/rounded
Chaenopsis						
alepidota M/F	same/same	* sail-like/even	* high/low	absent/absent	absent/absent	rounded/rounded
coheni M/F	same/same	* sail-like/even	* high/low	absent/absent	absent/absent	rounded/rounded
deltarrhis M/F	same/same	* sail-like/even	* high/low	absent/absent	absent/absent	rounded/rounded
limbaughi M/F	same/same	* sail-like/even	* high/low	absent/absent	absent/absent	pointed/pointed
ocellata M/F	same/same	* sail-like/even	* high/low	absent/absent	absent/absent	pointed/pointed
resh M/F	same/same	* sail-like/even	* high/low	absent/absent	absent/absent	rounded/rounded
roseola M/F	same/same	even/even	low/low	absent/absent	absent/absent	rounded/rounded
schmitti M/F	same/same	* sail-like/even	* high/low	absent/absent	absent/absent	rounded/rounded
new species. M/F	same/same	* sail-like/even	* high/low	absent/absent	absent/absent	rounded/rounded
Cirriemblemaria						
lucasana M/F	same/same	even/even	* moderate/low	* moderate/thin	* slight/deep	* rounded/pointed
Coralliozetus						
angelicus M/F	* few/many	* high/even	* moderate/low	* thin/absent	* slight/deep	* rounded/pointed
boehlkei M/F	* few/many	* high/even	* moderate/low	* thin/absent	* slight/deep	* rounded/pointed
cardonae M/F	* few/many	* high/anterior elevated	* moderate/low	* thin/absent	* slight/deep	* rounded/pointed
micropes M/F	* few/many	* sail-like/even	* high/low	* thin/absent	* slight/deep	* rounded/pointed
rosenblatti M/F	* few/many	* spike-like/anterior elevated	* moderate/low	* thin/absent	* slight/deep	* rounded/pointed
springeri M/F	* few/many	* high/anterior elevated	* moderate/low	* thin/absent	* slight/deep	* rounded/pointed
Ekemblemaria						
myersi M/F	same/same	even/even	low/low	thin/thin	absent/absent	rounded/rounded
nigra M/F	same/same	even/even	low/low	thin/thin	absent/absent	rounded/rounded

Emblemaria						
atlantica M/F	same/same	* sail-like/even	* high/low	absent/absent	slight/slight	* rounded/pointed
caldwelli M/F	same/same	* low sail/even	* high/low	absent/absent	* moderate/deep	* rounded/pointed
caycedoi M/F	same/same	sail-like/sail-like	* high/moderate	thin/thin	absent/absent	rounded/rounded
diphyodontis M/F	same/same	* sail-like/even	* high/low	* flag-like/absent	absent/absent	* rounded/pointed
hudsoni M/F	same/same	* sail-like/anterior elevated	* high/low	* flag-like/absent	absent/absent	* rounded/pointed
hyltoni M/F	same/same	thread-like/thread-like	long thread/long thread	absent/absent	slight/slight	* rounded/pointed
hypacanthus M/F	same/same	* sail-like/anterior elevated	* high/low	* flag-like/absent	* absent/slight	* rounded/pointed
nivipis M/F	same/same	* sail-like/anterior threadlike	* high/moderate	absent/absent	slight/slight	* rounded/pointed
pandionis M/F	same/same	* sail-like/anterior elevated	* high/low	absent/absent	slight/slight	* rounded/pointed
piratica M/F	same/same	* sail-like/anterior threadlike	* high/moderate	* flag-like/absent	absent/absent	* rounded/pointed
piratula M/F	same/same	* low sail/anterior elevated	* high/low	*thin/absent	* slight/moderate	* rounded/pointed
walkeri M/F	same/same	* sail-like/anterior elevated	* high/low	* flag-like/absent	absent/absent	* rounded/pointed
Emblemariopsis						
bahamensis M/F	same/same	even/even	low/low	absent/absent	slight/slight	pointed/pointed
diaphana M/F	same/same	even/even	low/low	absent/absent	slight/slight	pointed/pointed
leptocirris M/F	same/same	even/even	low/low	absent/absent	moderate/moderate	rounded/rounded
occidentalis M/F	same/same	anterior elevated/anterior elevated	low/low	absent/absent	slight/slight	pointed/pointed
pricei M/F	same/same	even/even	low/low	absent/absent	slight/slight	rounded/rounded
randalli M/F	same/same	even/even	low/low	absent/absent	slight/slight	rounded/rounded
signifera M/F	same/same	spike-like/spike-like	moderate spike/moderate spike	absent/absent	deep/deep	* rounded/pointed
Hemiemblemaria						
simulus M/F	same/same	even/even	low/low	absent/absent	slight/slight	rounded/rounded
Lucayablennius						
zingaro M/F	same/same	even/even	low/low	absent/absent	absent/absent	rounded/rounded
Mccoskerichthys						
sandae M/F	same/same	even/even	low/low	absent/absent	absent/absent	rounded/rounded
Neoclinus						
blanchardi M/F	same/same	even/even	* moderate/low	absent/absent	slight/slight	rounded/rounded
stephensae M/F	same/same	even/even	low/low	absent/absent	slight/slight	rounded/rounded
uninotatus M/F	same/same	even/even	* moderate/low	absent/absent	slight/slight	rounded/rounded
Protemblemaria						
bicirris M/F	same/same	even/even	low/low	thin/thin	slight/slight	rounded/rounded
perla M/F	same/same	even/even	low/low	thin/thin	slight/slight	rounded/rounded
punctata MF	same/same	* even/anterior elevated	low/low	thin/thin	slight/slight	rounded/rounded

	M-IVf. Pelvic fin rays	C-Ia. Head	C-Ib. Lower jaw	C-Ic. Branchiostegal membrane	C-IIa. Lateral body	C-IIb. Abdomen
Acanthemblemaria						
aspera M/F	incised/incised	* DM/FM	none/none	* DM/FM	* DM/FM	* DM/FM
atrata M/F	incised/incised	DM/DM	* DM/FM(bands)	DM/DM	DM/DM	DM/DM
balanorum M/F	incised/incised	DM/DM	DM/DM	* DM/FM	DM/DM	DM/DM
betinensis M/F	incised/incised	* DM/FM	* DM/FM	* DM/FM	* DM/FM	DM/DM
castroi M/F	incised/incised	DM/DM	* DM/FM(bands)	* DM/FM(bands)	DM/DM	* DM/FM(bands)
chaplini M/F	incised/incised	* DM/FM	* DM/FM	* DM/FM	* DM/FM	* DM/FM
crockeri M/F	incised/incised	* DM/FM	DM/DM	* DM/FM	* DM(spots)/FM(blotches)	* DM/FM
exilispinis M/F	incised/incised	DM/DM	DM/DM	DM/DM	DM/DM	FM/FM
greenfieldi M/F	incised/incised	DM/DM	DM/DM	* DM/FM	* DM/FM	DM/DM
hancocki M/F	incised/incised	* DM/FM	* DM/FM(bands)	* DM/FM	DM(spots)/DM(spots)	* DM/FM
harpezi M/F	incised/incised	FM/FM	none/none	* DM(elongate mark)/ none	none/none	* DM(patch)/none
hastingsi M/F	incised/incised	* DM/FM	* DM/FM(bands)	* DM/FM	* DM/FM	* DM/FM
macrospilus M/F	incised/incised	* DM/FM	* DM/FM(bands)	* DM/FM	* DM/FM	* DM/FM
mangognatha M/F	incised/incised	DM/DM	* DM/FM(bands)	* DM/FM	FM/FM	FM/FM
maria M/F	incised/incised	DM/DM	bands/bands	DM/DM	bands/bands	DM/DM
medusa M/F	incised/incised	* DM/FM(bands)	* DM/FM(bands)	* DM/FM	* DM/FM	DM/DM
paula M/F	incised/incised	FM/FM	none/none	* DM/FM	* FM/none to very few	FM/FM
rivasi M/F	incised/incised	DM/FM	* DM/FM	* DM/FM	* DM/FM	* DM/FM
spinosa M/F	incised/incised	* DM/FM	bands/bands	* DM/FM	* DM/FM	DM/DM
stephensi M/F	incised/incised	DM/DM	* DM/FM(bands)	* DM/FM	* DM/FM	* DM/FM
Chaenopsis						
alepidota M/F	incised/incised	* DM/FM	* DM/none	* DM/FM	* DM/FM	* DM/none
coheni M/F	incised/incised	* DM/FM	* FM/FM(bands)	* DM/FM	* DM/FM	* DM/FM
deltarrhis M/F	incised/incised	* DM/FM	* DM/FM(bands)	* DM/FM	DM/DM	* FM/none
limbaughi M/F	incised/incised	* DM/FM	* FM/FM(bands)	* DM/FM	* FM/none to very few	* FM/none
ocellata M/F	incised/incised	* DM/FM	* DM/FM	* DM/FM	* DM/FM	* DM/FM
resh M/F	incised/incised	* DM/FM	* DM/FM(bands)	* DM/FM	* DM/FM(bands)	* DM/FM
roseola M/F	incised/incised	* DM/FM	* DM/FM(bands)	none/none	blotches/blotches	none/none
schmitti M/F	incised/incised	* DM/FM	* DM/FM(bands)	* DM/FM	FM/FM	none/none
new species. M/F	incised/incised	* DM/FM	* DM/FM(bands)	dark mark/dark mark	DM/DM	FM/FM
Cirriemblemaria						
lucasana M/F	incised/incised	* FM(mask)/FM(single line)	none/none	none/none	none/none	none/none
Coralliozetus						
angelicus M/F	incised/incised	* DM/spots	* DM/FM(bands)	* DM/FM(bands)	* DM/none to few	* DM/none
boehlkei M/F	incised/incised	* DM(bands)/FM	* DM/FM(bands)	* DM(bands)/FM(spots)	* DM/none	* DM/none
cardonae M/F	incised/incised	* DM(mask)/FM(mask)	* DM/FM(bands)	* DM/FM(bands)	* FM/none	* DM/none
micropes M/F	incised/incised	* DM(bands)/FM(bands)	* DM/FM(bands)	* DM/FM(bands)	* DM/FM	* DM/none
rosenblatti M/F	incised/incised	* DM/FM(spots)	* DM/FM(bands)	* DM/FM(spots)	* DM/none	* DM/none
springeri M/F	incised/incised	* DM/none	* DM/FM(bands)	* DM/none	* FM/none	* DM/none
Ekemblemaria						
myersi M/F	incised/incised	DM/DM	* DM/FM(bands)	DM/DM	* DM(lined bars)/DM(bars)	DM/DM
nigra M/F	incised/incised	DM/DM	* DM/FM(bands)	DM/DM	DM/DM	DM/DM

Emblemaria						
atlantica M/F	* not incised/incised	* DM/FM	* DM(bands)/FM(bands)	* DM/FM	* DM/FM(bands)	* DM/FM
caldwelli M/F	incised/incised	* DM/FM	* DM/FM	* FM/none	* DM/FM to none	* DM/FM
caycedoi M/F	* not incised/incised	* DM/FM	* DM/FM	* DM/FM	* DM/FM(mottled)	FM/FM
diphyodontis M/F	* not incised/incised	* DM/FM(mottled)	* DM/FM(bands)	* DM/FM	* DM/FM	* DM/FM
hudsoni M/F	* not incised/incised	* DM/FM	* DM/FM(bands)	* DM/FM	* DM/FM	* DM/FM
hyltoni M/F	incised/incised	* DM/FM(mottled)	* DM/FM(bands)	* DM/FM	* DM/FM to none	* DM/none
hypacanthus M/F	* not incised/incised	* DM/FM(mottled)	* DM/FM(bands)	* DM/FM	* DM/FM(mottled)	* DM/FM
nivipis M/F	* not incised/incised	* DM/FM	* DM/FM(bands)	* DM/FM	* DM/FM(blotches)	* DM/FM
pandionis M/F	* not incised/incised	* DM/FM	* DM/FM(bands)	* DM/FM	* DM/FM(spots)	* DM/FM
piratica M/F	* not incised/incised	* DM/FM(mottled)	* DM/FM(bands)	* DM/FM	* DM(blotches)/FM(flecks)	* DM/none
piratula M/F	incised/incised	* DM/FM	* DM/none	* DM/none	* DM/FM to none	* DM/none
walkeri M/F	* not incised/incised	* DM/FM	* FM(bands)/DM(bands)	* DM(banda)/FM(bands)	blotches/blotches	none/none
Emblemariopsis						
bahamensis M/F	incised/incised	* DM/FM	* DM/FM	* DM/none	* FM/none	* FM/none
diaphana M/F	incised/incised	* DM/FM	* DM/none	* DM/FM(spots)	* FM(spots)/none	* DM/none
leptocirris M/F	incised/incised	* Dm/FM	* DM/FM	* DM/FM	* DM/FM or none	* FM/none
occidentalis M/F	incised/incised	* DM/none	* DM/none	* DM/none	* DM/FM	* FM/none
pricei M/F	incised/incised	* DM/none	* DM/FM(bands)	* DM/none	* DM/FM	none/none
randalli M/F	incised/incised	* DM/FM	FM/FM	FM/FM	* FM/none or spots	none/none
signifera M/F	incised/incised	* DM/FM	* DM/FM	* DM/FM(spots)	* FM/none	* DM/FM(blotch)
Hemiemblemaria						
simulus M/F	incised/incised	band/band	none/none	none/none	band/band	none/none
Lucayablennius						
zingaro M/F	incised/incised	none/none	none/none	none/none	* FM(along vent)/none	none/none
Mccoskerichthys						
sandae M/F	incised/incised	DM/DM	FM/FM	DM/DM	DM/DM	DM/DM
Neoclinus						
blanchardi M/F	incised/incised	DM/DM	DM/DM	DM/DM	DM/DM	DM/DM
stephensae M/F	incised/incised	DM/DM	* DM/FM(bands)	* DM/FM	* DM/FM(bands)	DM/DM
uninotatus M/F	incised/incised	DM/DM	DM/DM	DM/DM	DM/DM	DM/DM
Protemblemaria						
bicirris M/F	incised/incised	DM/DM	* DM/FM(bands)	* DM/FM(bands)	DM(saddles)/DM(saddles)	DM/DM
perla M/F	incised/incised	DM/DM	* DM(bands)/FM(bands)	DM/DM	FM saddles/FM saddles	DM/DM
punctata MF	incised/incised	DM/DM	* DM/FM(bands)	* DM/FM(bands)	DM/DM	DM/DM

	C-IIc. Anterior of pelvic fin	C-IIIa. Dorsal fin: anterior	C-IIIb. Dorsal fin: spot	C-IIIc. Dorsal fin: posterior	C-IIId. Dorsal fin: clear windows
Acanthemblemaria					
aspera M/F	same/same	* DM/FM	* III-IV/none	FM(none distally)/FM(none distally)	absent/absent
atrata M/F	same/same	DM/DM	I-II/I-II	FM(none distally)/FM(none distally)	absent/absent
balanorum M/F	same/same	DM/DM	none/none	FM/FM	absent/absent
betinensis M/F	same/same	* DM/FM	swath I-IV/swath I-IV	FM(none distally)/FM(none distally)	absent/absent
castroi M/F	same/same	* DM/FM	* I-II/none	FM(none distally)/FM(none distally)	absent/absent
chaplini M/F	same/same	* DM/FM	I-III/I-III	FM(none distally)/FM(none distally)	absent/absent
crockeri M/F	same/same	* DM/FM	none or I-II/none or I-II	FM(none distally)/FM(none distally)	absent/absent
exilispinis M/F	same/same	DM/DM	II-III/II-III	FM(none distally)/FM(none distally)	absent/absent
greenfieldi M/F	same/same	DM/DM	I-III or IV/I-III or IV	FM(none distally)/FM(none distally)	absent/absent
hancocki M/F	same/same	* DM/FM	I-II/I-II	FM(none distally)/FM(none distally)	absent/absent
harpezi M/F	same/same	none/none	none/none	FM/FM	absent/absent
hastingsi M/F	same/same	* DM/FM	none/none	FM(none distally)/FM(none distally)	absent/absent
macrospilus M/F	same/same	* DM/FM	I-III/I-III	FM(none distally)/FM(none distally)	absent/absent
mangognatha M/F	same/same	DM/DM	* I-II/none	FM(none distally)/FM(none distally)	absent/absent
maria M/F	same/same	DM/DM	none/none	FM(none distally)/FM(none distally)	absent/absent
medusa M/F	same/same	* DM/FM	I-III or II-IV/I-III or II-IV	FM(none distally)/FM(none distally)	absent/absent
paula M/F	same/same	FM/FM	none/none	FM(none distally)/FM(none distally)	absent/absent
rivasi M/F	same/same	* DM/FM	* I-II/I-II(smaller)	FM(none distally)/FM(none distally)	absent/absent
spinosa M/F	same/same	DM/DM	none/none	FM(none distally)/FM(none distally)	absent/absent
stephensi M/F	same/same	* DM/FM	I-II/I-II	FM(none distally)/FM(none distally)	absent/absent
Chaenopsis					
alepidota M/F	same/same	* DM/FM	I-II/I-II	* DM/FM	absent/absent
coheni M/F	same/same	* DM/FM	none/none	* DM(distal band)/none	absent/absent
deltarrhis M/F	same/same	* DM/FM(bands)	* none/III-IV	* DM/FM	absent/absent
limbaughi M/F	same/same	* DM/FM	* I-II/I-II(smaller)	FM/FM	absent/absent
ocellata M/F	same/same	* DM/FM	I-II/I-II	* DM/FM(diagonal band)	absent/absent
resh M/F	same/same	* DM/FM(spots)	* II-III/none	* spots(rows)/spots(diagonal bands)	absent/absent
roseola M/F	same/same	* DM/none	none/none	* FM/none	absent/absent
schmitti M/F	same/same	* DM/FM	* I-III/none	* FM distal third/none	absent/absent
new species. M/F	same/same	* DM/FM	* none/III-IV	FM/FM	absent/absent
Cirriemblemaria					
lucasana M/F		* DM/FM	* II-III/none	* DM/none	absent/absent
Coralliozetus					
angelicus M/F	same/same	* DM/none	* I-II/none	* DM/none	absent/absent
boehlkei M/F	same/same	* DM/ FM to none	none/none	* FM(bands)/none	absent/absent
cardonae M/F	same/same	* DM/FM to none	none/none	* FM/none	absent/absent
micropes M/F	same/same	* DM/FM	* none/I-II	* DM/FM	absent/absent
rosenblatti M/F	same/same	* DM/FM	* I-II/none	* FM(bands)/none	absent/absent
springeri M/F	same/same	* DM/FM(I-II only)	none/none	* FM/none	absent/absent
Ekemblemaria					
myersi M/F	* DM/clear spot	DM/DM	none/none	* less clear area/more clear area	* absent/present
nigra M/F	* DM/clear spot	DM/DM	none/none	* less clear area/more clear area	* absent/present

Emblemaria					
atlantica M/F	same/same	* DM/FM	I-II/I-II)	* DM/FM	absent/absent
caldwelli M/F	same/same	* DM/FM	none/none	* DM/none	absent/absent
caycedoi M/F	same/same	* DM/FM	none/none	* FM/none	absent/absent
diphyodontis M/F	same/same	* DM/FM(streaks)	none/none	* DM/FM(mottled)	absent/absent
hudsoni M/F	same/same	* DM/FM(bands)	* I-II/ none	* FM/FM(bands)	absent/absent
hyltoni M/F	same/same	* DM/FM(bands)	none/none	* DM/none	absent/absent
hypacanthus M/F	same/same	* DM/FM(mottled & bands)	none/none	* DM/FM(bands)	absent/absent
nivipis M/F	same/same	* DM/FM(mottled)	* none/III-V	* DM/FM	absent/absent
pandionis M/F	same/same	* DM/FM(mottled & bands)	none/none	* DM/FM(bands)	absent/absent
piratica M/F	same/same	* DM(bands)/FM(bands)	* none/IV-V(botch)	* DM(bands)/FM(spots)	absent/absent
piratula M/F	same/same	* DM/FM	none/none	* FM/none	absent/absent
walkeri M/F	same/same	* DM/FM(bands)	none/none	* DM/FM(spots)	absent/absent
Emblemariopsis					
bahamensis M/F	same/same	* DM/none	none/none	* DM/none	absent/absent
diaphana M/F	same/same	* DM/FM	none/none	none/none	absent/absent
leptocirris M/F	same/same	* DM/FM	none/none	none/none	absent/absent
occidentalis M/F	same/same	* DM/FM	none/none	none/none	absent/absent
pricei M/F	same/same	* DM/FM	none/none	none/none	absent/absent
randalli M/F	same/same	* DM/FM	none/none	* FM/none	absent/absent
signifera M/F	same/same	* DM(none distally)/DM	none/none	* FM/none	absent/absent
Hemiemblemaria					
simulus M/F	same/same	DM/DM	IV-VII/IV-VII	FM(band)/FM(band)	absent/absent
Lucayablennius					
zingaro M/F	same/same	* DM(band)/FM	none/none	three spots/three spots	absent/absent
Mccoskerichthys					
sandae M/F	same/same	FM/FM	none/none	FM/FM	absent/absent
Neoclinus					
blanchardi M/F	same/same	DM/DM	I-II/I-II	DM/DM	absent/absent
stephensae M/F	same/same	* DM/FM(bands)	none/none	* DM/FM(mottled)	absent/absent
uninotatus M/F	same/same	DM/DM	I-II/I-II	DM/DM	absent/absent
Protemblemaria					
bicirris M/F	same/same	DM/DM	II-III/II-III	FM/FM	absent/absent
perla M/F	same/same	* DM(spots)/DM	II-III/II-III	FM/FM	absent/absent
punctata MF	same/same	*DM/DM(black I-II)	* II-III/none	FM/FM	absent/absent

	C-IIIe. Caudal fin	C-IIIf. Anal fin	C-IIIg. Posterior median fins: clear area	C-IIIh. Pectoral-fin base	C-IIIi. Pectoral fin	C-IIIj. Pectoral fin bands	C-IIIk. Pelvic fin
Acanthemblemaria							
aspera M/F	none/none	* DM/none	same/same	* DM/FM	* FM/none	none/none	* FM/none
atrata M/F	FM/FM	DM/DM	same/same	DM/DM	DM/DM	none/none	DM/DM
balanorum M/F	FM/FM	DM/DM	same/same	DM/DM	FM/FM	none/none	DM/DM
betinensis M/F	FM/FM	* DM/FM	same/same	FM/FM	DM/DM	none/none	DM/DM
castroi M/F	FM/FM	DM/DM	same/same	DM/DM	FM/FM	none/none	* DM/FM
chaplini M/F	none/none	* DM(band)/FM	same/same	* DM/FM	* DM/FM	none/none	* DM/FM
crockeri M/F	* FM/none	* DM(band)/FM	same/same	DM/DM	FM/FM	none/none	FM/FM
exilispinis M/F	none/none	FM(blotches)/FM(blotches)	same/same	FM/FM	FM/FM	none/none	FM/FM
greenfieldi M/F	none/none	DM/DM	same/same	DM/DM	FM/FM	none/none	FM/FM
hancocki M/F	none/none	* DM(bands)/FM(bands)	same/same	* DM(spot)/FM(spot)	* DM/FM	none/none	* DM/FM
harpezi M/F	none/none	FM/FM	same/same	none/none	none/none	none/none	* DM(patch)/FM(smaller)
hastingsi M/F	none/none	* DM(bands)/FM(bands)	same/same	* DM/FM	* DM/FM	none/none	* DM/FM
macrospilus M/F	none/none	* DM(bands)/FM(bands)	same/same	* DM/FM	* DM/FM	none/none	* DM/FM
mangognatha M/F	none/none	FM(band)/FM(band)	same/same	DM/DM	FM/FM	none/none	FM/FM
maria M/F	none/none	FM/FM	same/same	DM/DM	FM/FM	none/none	DM/DM
medusa M/F	none/none	* DM/FM	same/same	* DM/FM	FM/FM	none/none	FM/FM
paula M/F	none/none	none/none	same/same	FM/FM	DM/DM	none/none	DM/DM
rivasi M/F	none/none	* DM(bands)/FM(bands)	same/same	* DM/FM	* DM/FM	none/none	* DM/FM
spinosa M/F	none/none	FM/FM	same/same	* DM/FM	* DM/FM	none/none	* DM/FM
stephensi M/F	* DM/FM	* DM/FM	same/same	* DM/FM	* DM/FM	none/none	* DM/FM
Chaenopsis							
alepidota M/F	FM/FM	* DM/FM	same/same	* DM/FM	FM/FM	none/none	* DM/FM
coheni M/F	none/none	* DM(band)/FM	same/same	* DM/FM(spots)	* FM/none	none/none	* DM/none
deltarrhis M/F	FM/FM	FM(band)/FM(band)	same/same	* DM/FM	* FM/none	none/none	* FM/none
limbaughi M/F	FM/FM	* DM/FM(band)	same/same	* DM/FM	none/none	none/none	* FM/none
ocellata M/F	FM/FM	* DM/FM(band)	same/same	DM/DM	none/none	none/none	FM/FM
resh M/F	* FM/none	* DM/FM	same/same	* DM/FM	none/none	none/none	* FM/none
roseola M/F	FM/FM	* FM/none	same/same	none/none	none/none	none/none	FM/FM
schmitti M/F	none/none	* DM/none	same/same	* DM/FM	none/none	none/none	* FM/none
new species. M/F	none/none	FM(band)/FM(band)	same/same	FM/FM	none/none	none/none	* DM/FM
Cirriemblemaria							
lucasana M/F	none/none	* DM/none	same/same	none/none	* FM/none	none/none	* none/two spots
Coralliozetus							
angelicus M/F	* FM/none	* DM/none	same/same	* DM/FM	* DM/FM	none/none	* DM/none
boehlkei M/F	* DM(bands)/FM(bands)	* DM/FM(spots)	same/same	* DM/FM(spots)	* DM(bands)/FM(bands)	present/present	* DM/none
cardonae M/F	* FM/none	FM/FM	same/same	* DM/FM(blotches)	* FM/none	none/none	* DM/none
micropes M/F	* DM/FM	* DM/FM(spots)	same/same	* DM/FM(spots)	* DM/FM(bands)	* none/present	* DM/none
rosenblatti M/F	* FM(bands)/fewer(bands)	* DM/FM(spots)	same/same	* DM/FM(spots)	* DM(bands)/FM(bands)	present/present	* DM/none
springeri M/F	* FM/none	FM(spots)/FM(spots)	same/same	* DM/FM	* FM/none	none/none	* DM/none
Ekemblemaria							
myersi M/F	clear/clear	DM/DM	* less/more	DM/DM	none/none	none/none	DM/DM
nigra M/F	clear/clear	DM/DM	* less/more	DM/DM	none/none	none/none	DM/DM

Emblemaria							
atlantica M/F	* DM(bands)/FM(bands)	* DM/FM(bars)	same/same	* DM/FM	none/none	none/none	* DM/none
caldwelli M/F	* FM/none	* DM/none	same/same	* DM/FM	none/none	none/none	* FM/none
caycedoi M/F	none/none	* DM/none	same/same	* DM/FM	none/none	none/none	* DM/none
diphyodontis M/F	* DM/FM	* DM/FM(band)	same/same	* DM/FM	* DM/FM	none/none	* DM/none
hudsoni M/F	* FM/none	* DM/FM(bars)	same/same	* DM/FM	FM/FM	none/none	* DM/FM
hyltoni M/F	* FM/none	* DM/none	same/same	* DM/FM	none/none	none/none	* DM/none
hypacanthus M/F	* DM/FM	* DM/FM(bars)	same/same	* DM/FM	* FM/none	none/none	* DM/none
nivipis M/F	* DM/FM	* DM/FM(band)	same/same	* DM/FM	* FM/none	none/none	* DM/FM
pandionis M/F	* FM/FM(bands)	* DM/FM(band)	same/same	* DM/FM	* FM/none	none/none	* DM/FM
piratica M/F	none/none	* DM/FM(bars)	same/same	* DM/FM	* DM/FM	none/none	* DM/FM
piratula M/F	* FM/none	* DM/none	same/same	* DM/none	* FM/none	none/none	* DM/none
walkeri M/F	none/none	* DM/FM(bars)	same/same	FM/FM	none/none	none/none	* DM/none
Emblemariopsis							
bahamensis M/F	none/none	* FM/none	same/same	* DM/none	* DM/none	none/none	* DM/none
diaphana M/F	none/none	* DM(band)/none	same/same	* DM(spots)/FM(spots	* DM/none	none/none	* DM/none
leptocirris M/F	none/none	* DM(anteriorly)/FM(or non	same/same	* DM/none	* DM/none	none/none	* DM/none
occidentalis M/F	none/none	* DM/none	same/same	* FM/none	* FM/none	none/none	* DM/none
pricei M/F	none/none	* DM/FM	same/same	* DM/none	* DM/FM(none)	none/none	* DM/none
randalli M/F	none/none	* DM/FM	same/same	* FM/none	* FM/none	none/none	none/none
signifera M/F	* FM/none	* DM/FM	same/same	* FM/none	* FM/none	none/none	* FM/none
Hemiemblemaria							
simulus M/F	none/none	FM/FM	same/same	band/band	none/none	none/none	none/none
Lucayablennius							
zingaro M/F	none/none	* DM(band)/FM	same/same	none/none	none/none	none/none	none/none
Mccoskerichthys							
sandae M/F	none/none	FM/FM	same/same	DM/DM	DM/DM	none/none	FM/FM
Neoclinus							
blanchardi M/F	FM/FM	DM/DM	same/same	DM/DM	DM/DM	none/none	DM/DM
stephensae M/F	FM/FM	*DM(band)/FM(mottled)	same/same	* DM/FM	* DM/FM	none/none	* DM/FM
uninotatus M/F	FM/FM	DM/DM	same/same	DM/DM	FM/FM	none/none	DM/DM
Protemblemaria							
bicirris M/F	none/none	DM/DM	same/same	DM/DM	FM/FM	none/none	DM/DM
perla M/F	FM/FM	* DM/FM	same/same	DM/DM	* DM/FM	none/none	* DM/FM
punctata MF	FM/FM	DM/DM	same/same	DM/DM	FM/FM	none/none	DM/DM
